# ZFP36L1 and ZFP36L2 control LDLR mRNA stability via the ERK–RSK pathway

**DOI:** 10.1093/nar/gku652

**Published:** 2014-08-08

**Authors:** Shungo Adachi, Masae Homoto, Rikou Tanaka, Yusaku Hioki, Hiroshi Murakami, Hiroaki Suga, Masaki Matsumoto, Keiichi I. Nakayama, Tomohisa Hatta, Shun-ichiro Iemura, Tohru Natsume

**Affiliations:** 1 Molecular Profiling Research Center for Drug Discovery (molprof), National Institute of Advanced Industrial Science and Technology (AIST), Tokyo 135-0064, Japan; 2 Galaxy Pharma Inc., Akita 010-0951, Japan; 3 Department of Life Sciences, Graduate School of Arts and Sciences, The University of Tokyo, Meguro-ku, Tokyo 153-8904, Japan; 4 Department of Chemistry, Graduate School of Science, The University of Tokyo, 7-3-1 Bunkyo-ku, Tokyo 113-0033, Japan; 5 Department of Molecular and Cellular Biology, Medical Institute of Bioregulation, Kyushu University, Fukuoka 812-8582, Japan

## Abstract

Low-density lipoprotein receptor (LDLR) mRNA is unstable, but is stabilized upon extracellular signal-regulated kinase (ERK) activation, possibly through the binding of certain proteins to the LDLR mRNA 3′-untranslated region (UTR), although the detailed mechanism underlying this stability control is unclear. Here, using a proteomic approach, we show that proteins ZFP36L1 and ZFP36L2 specifically bind to the 3′-UTR of LDLR mRNA and recruit the CCR4-NOT-deadenylase complex, resulting in mRNA destabilization. We also show that the C-terminal regions of ZFP36L1 and ZFP36L2 are directly phosphorylated by p90 ribosomal S6 kinase, a kinase downstream of ERK, resulting in dissociation of the CCR4-NOT-deadenylase complex and stabilization of LDLR mRNA. We further demonstrate that targeted disruption of the interaction between LDLR mRNA and ZFP36L1 and ZFP36L2 using antisense oligonucleotides results in upregulation of LDLR mRNA and protein. These results indicate that ZFP36L1 and ZFP36L2 regulate LDLR protein levels downstream of ERK. Our results also show the usefulness of our method for identifying critical regulators of specific RNAs and the potency of antisense oligonucleotide-based therapeutics.

## INTRODUCTION

Messenger RNA (mRNA) turnover plays a key role in the regulation of protein levels. This regulation is achieved through *cis*-regulatory elements, including adenosine and uridine (AU)-rich elements (AREs) residing in the 3′-untranslated regions (UTRs) of mRNAs. AREs are present in many translationally repressed and unstable mRNA species and play a role in 5–8% of all mRNAs (1). Destabilization of ARE-containing mRNAs is accomplished through their interaction with ARE-binding proteins (ARE-BPs). Many ARE-BPs, including hnRNPD, KHSRP, DHX36 and ZFP36 family proteins have been identified (2); however, predicting which protein can bind to a specific ARE is still very difficult (2).

The low-density lipoprotein (LDL) receptor (LDLR) is a receptor for circulating LDL and has a critical role in removing LDL from blood (3). A high blood level of LDL cholesterol is a major risk factor for heart disease. Development of a drug to increase the amount of LDLR protein in the liver to lower LDL cholesterol is advanced and would be highly beneficial (3).

LDLR mRNA is known to be unstable, but is stabilized upon phorbol 12-myristate 13-acetate (PMA) treatment through the activation of extracellular signal-regulated kinase (ERK) (4). The 3′-UTR of LDLR mRNA, particularly the 1 kb 5′ region that contains three AREs (ARE1–3), and the proteins that bind this region are thought to be involved in this stabilization. Although some LDLR mRNA-binding proteins have been identified, the detailed mechanisms underlying the control of LDLR mRNA stability remain unknown (4,5), and the critical regulator of this control has not been identified.

Here, using a proteomic approach, we found that ZFP36L1 and ZFP36L2 bind specifically to the LDLR mRNA 3′-UTR region. ZFP36L1 and ZFP36L2 belong to the family of CCCH tandem zinc finger proteins (the ZFP36 family, which includes ZFP36, ZFP36L1 and ZFP36L2) (6). ZFP36 family proteins bind to AREs and trigger the degradation of several ARE-containing mRNAs, including PLK3 and vascular endothelial growth factor A (VEGFA) (7,8). We examined the role of ZFP36L1 and ZFP36L2 in LDLR mRNA stability using an RNAi-based knockdown method and we found that ZFP36L1 and ZFP36L2 destabilize LDLR mRNA. We also found that p90 ribosomal S6 kinase (RSK)1, a kinase downstream of ERK, directly phosphorylates the C-terminus of ZFP36L1 and inhibits the mRNA-destabilizing activity of ZFP36L1. From these results, we conclude that ZFP36L1 and ZFP36L2 regulate the levels of LDLR protein downstream of ERK.

We then tried to disrupt the interaction between LDLR-mRNA and ZFP36L1 and ZFP36L2 proteins using locked nucleic acid (LNA)- (9) modified antisense oligonucleotides. We were able to selectively disrupt the interaction between LDLR-mRNA and ZFP36L1 and ZFP36L2 without affecting interactions of ZFP36L1 and ZFP36L2 with other target mRNAs. This resulted in an increase in LDLR protein levels. Our results show the usefulness of our method for identifying regulators of specific mRNAs and also show the potency of antisense oligonucleotide-based therapeutics.

## MATERIALS AND METHODS

### Cell culture

HEK293T and HeLa cells were cultured at 37°C in Dulbecco's modified Eagle's medium supplemented with 10% fetal bovine serum. Hep3B cells were cultured at 37°C in minimal essential medium supplemented with nonessential amino acids and 10% fetal bovine serum.

### Preparation of bait RNAs

T7 tagged cDNA template was polymerase chain reaction (PCR) amplified and subjected to *in vitro* transcription using a MEGAscript T7 kit (Applied Biosystems). Amplified cRNA was purified with an RNeasy Mini Kit (Qiagen) and then subjected to Flag conjugation as described (10) with some modifications. Briefly, 60 μl of freshly prepared 0.1 M NaIO_4_ was added to 60 μl of 250 pmol cRNA, and the mixture was incubated at 0°C for 10 min. The 3′dialdehyde RNA was precipitated with 1 ml of 2% LiClO_4_ in acetone followed by washing with 1 ml acetone. The pellet was dissolved in 10 μl of 0.1 M sodium acetate, pH 5.2 and then mixed with 12 μl of 30 mM hydrazide–Flag peptide. The reaction solution was mixed at room temperature for 30 min. The resulting imine-moiety of the cRNA was reduced by adding 12 μl of 1 M NaCNBH_3_, and then incubated at room temperature for 30 min. The RNA was purified with an RNeasy Mini Kit (Qiagen). The regions of bait RNAs used for immunoprecipitation (IP) experiments are shown in Supplemental Table IV.

### Purification and analysis of RNA-binding protein

Purification and analysis of RNA-binding protein (RBP) were carried out as described (11) with some modifications. Briefly, 293T cells were lysed with lysis buffer [10 mM 4-(2-hydroxyethyl)-1-piperazineethanesulfonic acid (HEPES) (pH 7.5), 150 mM NaCl, 50 mM NaF, 1 mM Na_3_VO_4_, 5 μg/ml leupeptin, 5 μg ml aprotinin, 3 μg/ml pepstatin A, 1 mM phenylmethylsulfonyl fluoride (PMSF), 1 mg/ml digitonin] and cleared by centrifugation. The cleared lysate was incubated with indicated amounts of Flag-tagged bait RNA, antisense oligos and Flag-M2-conjugated agarose for 1 h. The agarose resin was then washed three times with wash buffer [10 mM HEPES (pH 7.5), 150 mM NaCl, 0.1% Triton X-100] and co-immunoprecipitated RNA and proteins were eluted with Flag elution buffer [0.5 mg/ml Flag peptide, 10 mM HEPES (pH 7.5), 150 mM NaCl, 0.05% Triton X-100]. The bait RNA associated proteins were digested with lysyl endopeptidase, and the resulting peptides were analyzed using a nanoscale liquid-chromatography tandem mass spectrometry (LC/MS/MS) system.

### Western blot analysis

Whole-cell lysates or immunoprecipitates were resolved by sodium dodecyl sulphate (SDS) polyacrylamide gel electrophoresis and then transferred onto Immobilon-P membranes (Millipore). The membranes were probed with the indicated antibodies and proteins of interest were visualized with horseradish peroxidase-conjugated mouse, rabbit or goat immunoglobulin G using ECL Plus (GE). Intensity of individual bands was quantified using Multi Gauge software (Fuji Photo Film).

### Quantitative reverse-transcription PCR

Total RNA was purified using the RNeasy Mini Kit (Qiagen). cDNA was synthesized using the High Capacity cDNA Reverse Transcription Kit (Invitrogen). Quantitative PCR (qPCR) was performed using Fast SYBR Green on a StepOnePlus system (Applied Biosystems). The following PCR primers were used: human β-actin: forward: 5′-TGGATCAGCAAGCAGGAGTATG-3′, reverse: 5′-GCATTTGCGGTGGACGAT-3′, human LDLR: forward: 5′-CCCGACCCCTACCCACTT-3′, reverse: 5′-AATAACACAAATGCCAAATGTACACA-3′, human PLK3: forward: 5′-CTGCGCCATGACTTCTTTACC-3′, reverse: 5′-GTCACGCAGCTGCTGATAGG-3′, human VEGFA: forward: 5′-CGAGGGCCTGGAGTGTGT-3′, reverse: 5′-CCGCATAATCTGCATGGTGAT-3′, Red Fluorescent Protein (RFP): forward: 5′-AGACCACCTACATGGCCAAGA-3′, reverse: 5′-CTCGTTGTGGGAGGTGATGTC-3′, Luc2: forward: 5′-ACGAGCACTTCTTCATCGTG-3′, reverse: 5′-CCTGGTAGCCCTTGTATTTGA-3′.

Half-lives of mRNAs were calculated by fitting an exponential decay curve to the mRNA levels determined at all time points.

### Expression constructs

3′-UTR regions of LDLR mRNA were cloned into pDEST12.2 (Invitrogen), which contains a 5′-RFP tag. 3′-UTR regions of β-actin mRNA were cloned into pDEST12.2 (Invitrogen), which contains a 5′-LUC2 tag. Human ZFP36, ZFP36L1 and ZFP36L2 open reading frames were cloned into pDEST12.2 (Invitrogen), which contains a 5′-MYC tag or a 5′-Flag tag, or into pDEST15 (Invitrogen).

### Antibodies

The following antibodies were used for IP and/or western blot analysis: anti-β-actin (#4970; Cell Signalling), anti-CDK9 (sc-13130; Santa Cruz), anti-CNOT1 (14276-1-AP; Protein Tech), anti-CNOT7 (H00029883; Abnova), anti-FLAG (M2; Sigma), anti-hnRNPD (Q14103; Millipore), anti-HA (1867423; Roche), anti-IGF2BP1 (sc-21026; Santa Cruz), anti-KHSRP (ab83291; Abcam), anti-LARP7 (LaRP7-101AP; FabGennix Inc.), anti-LDLR (AF2148; BD Biosciences), anti-Myc (9E10; Roche), anti-phospho-ERK (#9101s; Cell Signalling), anti-phospho-MAPKAPK2 (#3007s; Cell Signalling), anti-phospho-RSK (sc-17033; Santa Cruz), anti-ZFP36L1 (#2119; Cell Signalling), anti-phospho-S6 (#2211; Cell Signalling).

### Chemicals

Cells were treated with each chemical as described below. PMA (Sigma) was used at a final concentration of 100 ng/ml. U0126 (Cell Signalling) was used at a final concentration of 10 μM. BI-D1870 (Stemgent) was used at a final concentration of 20 μM. SL0101 (Millipore) was used at a final concentration of 75 μM. Actinomycin D (ActD) (Calbiochem) was used at a final concentration of 5 μg/ml.

### Isobaric tags for relative and absolute quantitation (iTRAQ)-based quantification of phosphopeptides

FLAG-tagged ZFP36L1 and ZFP36L2 were transiently expressed in 293T cells. These proteins were purified using anti-Flag (M2) agarose beads (Sigma) and subjected to in solution digestion by lysyl endopeptidase and trypsin. Digested peptide mixtures were labeled with iTRAQ reagents (114 for PMA-treated sample; 115 for PMA+U0126-treated sample; 116 for untreated sample) according to the manufacturer's instructions and then loaded onto Fe-charged Probond (Fe-IMAC) columns (Applied Biosystems) (12). Loading/washing buffer for Fe-IMAC columns was 0.1% trifluoroacetic acid, 60% acetonitrile. After washing, bound peptides were eluted with 1% phosphate. Peptides were analyzed using a nanoLC/MS/MS system (QSTAR Elite, AB/MDS-Sciex) and a nanoLC system (Paradigm MS2, Michrom BioResources). Peak lists were obtained from the script using Analyst QS 2.0. MASCOT searches were performed against IPI human ver.3.1.6.

### Short interfering RNA and antisense oligonucleotides

Short interfering RNAs (siRNAs) against human ZFP36L1 (Cat. No. HSS101104, HSS101101) and ZFP36L2 (Cat. No. HSS101105, HSS101102) and control siRNA (Cat. No. 12935-100) were purchased from Invitrogen. These siRNAs were transfected into cells using DarmaFECT 2 (Thermo Scientific) at a final concentration of 20 nM. All the antisense-oligonucleotides against human LDLR mRNA were fully LNA-modified and were purchased from Gene Design Inc.: Oligo-L1 (5′-AGATGAATAAA-3′), Oligo-L2 (5′-GCCTCCCAGAT-3′), Oligo-L3 (5′-CACTTAATAAA-3′), Oligo-L4 (5′-ATAATAACACA-3′), Oligo-L5 (5′-AGATGAAGAAA-3′), Oligo-L6 (5′-AGAATAATAGA-3′). These oligonucleotides were transfected into cells using DarmaFECT 2 (Thermo Scientific) at a final concentration of 80 nM.

### Analysis of direct phosphorylation

Wild-type and mutant GST-ZFP36L1 were expressed in *Escherichia coli* and purified using glutathione-sepharose (GE). Two micrograms of purified GST-ZFP36L1 (unedited from glutathione-sepharose beads) and 0.5 μg of purified kinase were incubated in a kinase assay buffer [25 mM Tris (pH 7.5), 10 mM MgCl_2_, 2 mM Na_3_VO_4_, 1 mM dithiothreitol (DTT), 1 mM adenosine triphosphate] at 30°C for 30 min with continuous mixing (total volume of 100 μl). Glutathione-sepharose beads were washed in wash buffer [10 mM HEPES (pH 7.5), 150 mM NaCl, 0.1% Triton X-100] three times. We then analyzed the phosphorylation of GST-ZFP36L1 using MS. We also examined the ability of GST-ZFP36L1 proteins to interact with CNOT using IP and western blot analysis.

### Cell-based DiI-LDL uptake assay

Hep3B cells were transfected with indicated oligos. Twenty-four hours after transfection, cells were treated with DiI-LDL (final concentration 1 μg/ml, Molecular Probes) for 1 h and then lysed in RIPA buffer (25 mM Tris–HCl pH 7.6, 150 mM NaCl, 1% NP-40, 1% sodium deoxycholate, 0.1% SDS). DiI-LDL fluorescence (excitation/emission at 530/590 nm) was read on an Infinite 200 (Tecan) and protein levels were quantified using a BCA Protein Assay Kit (Thermo). For data analysis, the ratio of DiI-LDL fluorescence/protein concentration was used to normalize DiI-LDL uptake into cells.

## RESULTS

**Figure 1.**
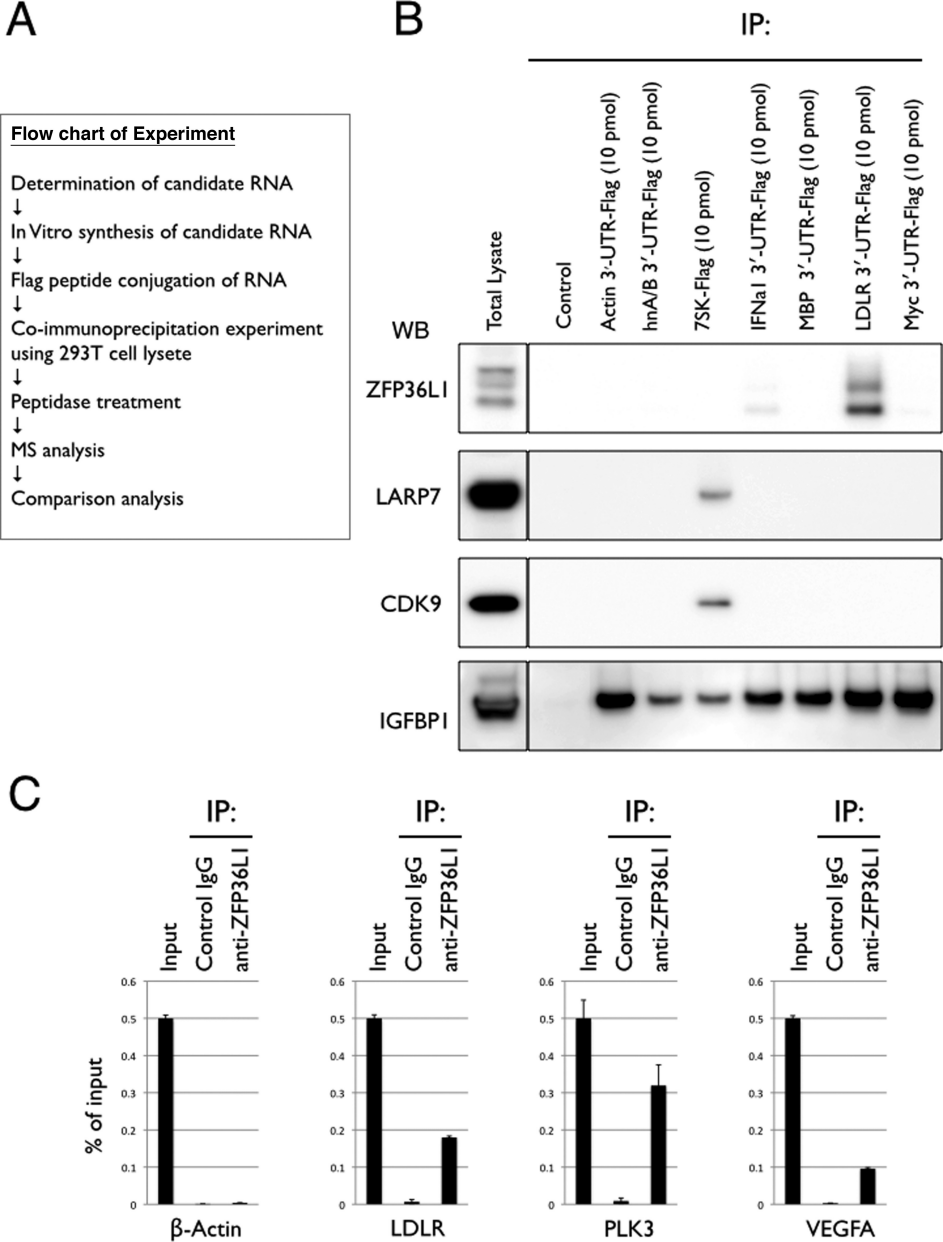
Identification of bait-specific RBPs. (**A**) Flow chart summarizing the experiment. (**B**) Confirmation of our results by western blot analysis. 293T cells were lysed in lysis buffer and the cleared lysates were subjected to IP with anti-Flag antibody in combination with the indicated bait RNAs. Co-immunoprecipitated RNA and proteins were eluted using the Flag-peptide and were then subjected to western blot analysis using the indicated antibodies. Five percent of the initial amount of cleared 293T lysate was loaded as total lysate. An IP experiment without bait RNA was performed as a control. (**C**) Confirmation of the endogenous interaction between ZFP36L1 and LDLR mRNA. 293T cells were lysed in lysis buffer and the cleared lysates were subjected to IP with control or anti-ZFP36L1 antibody. Total RNA and co-immunoprecipitated RNA were extracted, and quantitative reverse-transcription (RT)-PCR (qPCR) was performed using primers specific to LDLR, PLK3, VEGFA and β-actin mRNAs. Results are shown as% of input. The data are representative of at least three independent experiments. Error bars show standard deviation of the mean.

To identify the critical protein controlling the stability of LDLR mRNA, we first developed the method of Flag-peptide-tagging the 3′-end of *in vitro* transcribed RNA (Supplementary Figure S1A and B; see Experimental Procedures). We then validated whether Flag-peptide-tagged RNA can be used to co-immunoprecipitate its binding protein, using HA-tagged-MS2 and a Flag-peptide-tagged-RNA that contains an MS2-binding site (13) (Supplementary Figure S1C). We found that Flag-peptide-tagged RNA can be used for co-immunoprecipitation of its binding protein (Supplementary Figure S1D). Next, we hypothesized that the critical protein controlling LDLR mRNA stability would bind specifically to its 3′-UTR region, but would not bind to stable mRNAs or unstable mRNAs that are not stabilized by PMA treatment. We then selected seven bait RNAs, including LDLR mRNA, five stable RNAs (β-actin mRNA, IFNA1 mRNA, MBP mRNA, hnRNP A2/B1 mRNAs and 7SK RNA) and one very unstable mRNA, c-Myc, which is not stabilized by PMA treatment (Table S1). We synthesized these RNAs *in vitro* and conjugated a Flag-peptide to their 3′-ends. We performed an IP experiment using these seven bait RNAs and a 293T cell lysate. The co-immunoprecipitated proteins were eluted using the Flag peptide, and then digested with lysyl endopeptidase, and all peptides obtained were directly analyzed by MS (Figure 1A). For each RNA, we conducted two independent IP experiments and performed MS analysis in duplicate to obtain four sets of data. We identified about 400 kinds of peptides derived from ∼150 proteins (Table S2). Approximately 25% of these proteins, including IGF2BP1, were common to all the RNA baits. We then extracted the LDLR mRNA-specific binding proteins that were only identified in all four MS analyses of LDLR samples and found ZFP36L1 and ZFP36L2 as proteins that bind specifically to the LDLR mRNA 3′-UTR (ARE1–3) (Table S3). Using this method, we also found well-known 7SK RBPs, including CDK9 and LARP7 as 7SK-Flag specific binding porteins (14) (Table S4). This result demonstrates the accuracy of our strategy. We confirmed the interactions of bait RNAs and their specific binding proteins by western blotting (Figure 1B). To further confirm the endogenous interaction between LDLR mRNA and ZFP36L1, we performed a co-immunoprecipitation experiment using the antibody against ZFP36L1 and 293T cell lysate. We found that endogenous ZFP36L1 interacts with LDLR mRNA, and also with PLK3 and VEGFA mRNAs, previously identified ZFP36L1-interacting mRNAs (7,8). ZFP36L1 did not interact with β-actin mRNA (Figure 1C).

### ZFP36L1 and ZFP36L2 bind to LDLR mRNA

**Figure 2.**
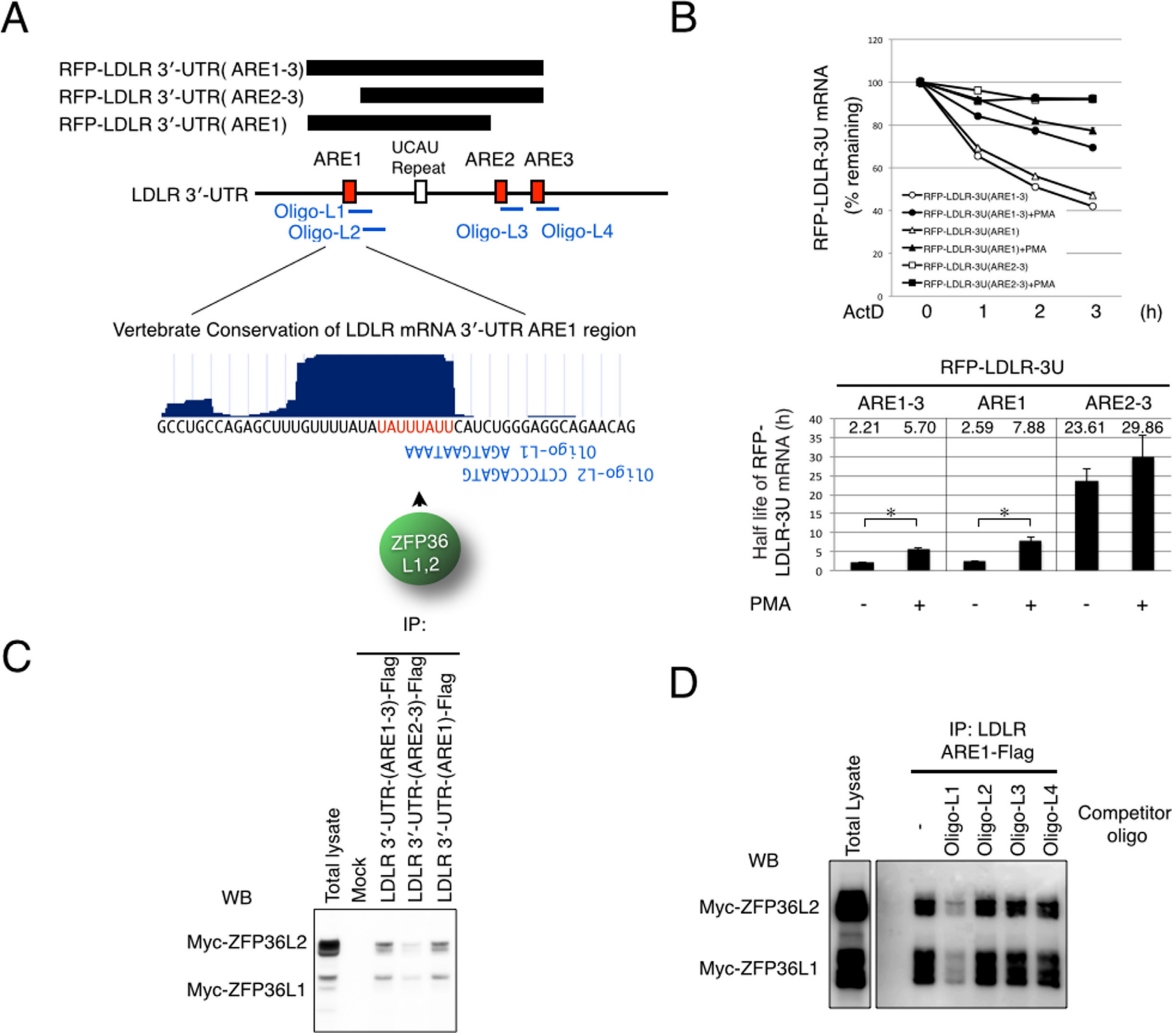
Domain analysis of the 3′-UTR of LDLR mRNA. (**A**) Schematic representation of the 3′-UTR of LDLR mRNA. Black bars indicate the regions used for ActD chase and IP experiments. AREs 1–3 are indicated by red boxes and the UCAU repeat is indicated by the white box. Designed oligonucleotides are indicated by blue lines. Conservation around the LDLR ARE1 region is shown in the blue graph, created using Vertebrate Multiz Alignment & PhastCons Conservation (28 species), as extracted from the UCSC Human Genome Browser (http://genome.ucsc.edu/). The consensus ZFP36L1- and ZFP36L2-binding sequence is colored in red. Sequences of the designed oligonucleotides, L1 and L2, are indicated. (**B**) RFP-tagged LDLR 3′-UTR constructs were expressed in 293T cells, and cells were then subjected to ActD chase experiments. Twenty-four hours after transfection, 293T cells were treated with ActD and PMA as indicated (PMA treatment commenced 10 min after ActD treatment). qPCR was performed using RFP and Luc2-specific primers. Results were normalized to the levels of Luc2 mRNA. The data are representative of at least three independent experiments. mRNA half-lives were calculated by fitting an exponential decay curve to all time points. Figures in the graph indicate the mean value of the mRNA decay half-lives. Error bars show standard deviation of the mean. *P*-values were calculated using Student's *t*-test. *P < 0.002; *n* = 3 for each group. (**C**) 293T cells were transfected with Myc-tagged ZFP36L1 and ZFP36L2. Cells were then lysed with lysis buffer and the cleared lysates were subjected to IP with the indicated LDLR 3′-UTR bait RNAs. Immunoprecipitated proteins were subjected to western blot analysis with the anti-Myc antibody. (**D**) 293T cells were transfected with Myc-tagged ZFP36L1 and ZFP36L2. Cells were then lysed with lysis buffer and the cleared lysates were subjected to IP with the indicated LDLR 3′-UTR bait RNAs and the indicated oligos. Immunoprecipitated proteins were subjected to western blot analysis with the anti-Myc antibody. These data are representative of at least three independent experiments.

ZFP36L1 and ZFP36L2 are known as proteins that bind to a certain type of ARE that contains the sequence UAUUUAUU, causing destabilization of target mRNAs. The LDLR mRNA 3′-UTR contains three AREs (ARE1–3) (4); ARE1 and ARE2 are comprised of the UAUUUAUU sequence. We investigated the region responsible for LDLR mRNA instability and PMA-mediated stabilization (PMA is an activator of ERK) in an ActD chase experiment. After transfection of 293T cells with the reporter constructs, RFP-LDLR-3′-UTR-ARE1–3, RFP-LDLR-3′-UTR-ARE2–3 or RFP-LDLR-3′-UTR-ARE1, we examined the stability of the reporter mRNA and the effect of PMA on stability using quantitative reverse-transcription (RT)-PCR (qPCR) analysis. We also calculated the half-life of each RFP-reporter mRNA (Figure 2A and B). We found that the ARE1-containing region is not only responsible for LDLR mRNA instability, but is also responsible for PMA-mediated stabilization of LDLR mRNA.

We then investigated which regions of the LDLR mRNA 3′-UTR are responsible for binding to ZFP36L1 and ZFP36L2 using several Flag-peptide-tagged 3′-UTR fragments of the LDLR mRNA, including LDLR-3′-UTR-ARE1–3, LDLR-3′-UTR-ARE2–3 and LDLR-3′-UTR-ARE1. We found that ZFP36L1 and ZFP36L2 predominantly bind to the ARE1 region, and only modestly bind to the ARE2–3 region, despite the presence of the UAUUUAUU sequence (Figure 2A and C).

To confirm the binding of ZFP36L1 and ZFP36L2 to the UAUUUAUU sequence of ARE1, we used LNA-modified antisense oligonucleotides. First, we designed LNA-modified 11-base oligonucleotides (Oligo-L1–L4). Oligo-L1 was complementary to the evolutionary conserved UAUUUAUU sequence in the ARE1 region, and to an LDLR gene-specific sequence located immediately 3′ to the UAUUUAUU sequence (Figure 2A and Supplementary Figure S2A). Oligo-L2 was designed to interact with the 3′-flanking region of the UAUUUAUU sequence, but not with UAUUUAUU itself (Figure 2A). Oligo-L3 and -L4 were designed to be complementary to the LDLR mRNA ARE-2 and -3 regions, respectively (Figure 2A). We then performed co-immunoprecipitation experiments with or without Oligo-L1, -L2, -L3 and -L4 to examine the ability of oligonucleotides to disrupt the *in vitro* interaction between LDLR mRNA and ZFP36L1 or ZFP36L2. We found that interaction was clearly blocked by Oligo-L1, but was not affected by Oligo-L2, -L3 or -L4 (Figure 2D). On the other hand, Oligo-L1 did not disrupt the interaction between LDLR mRNA and KHSRP, hnRNPD or hnRNPI, which have recently been identified as LDLR mRNA-destabilizing proteins (Supplementary Figure S2B). Furthermore, Oligo-L1 had no effect on the interaction between ZFP36L1 and the 3′-UTR regions of VEGFA or PLK3 mRNAs, recently identified as target mRNAs of ZFP36L1 (7,8), (Supplementary Figure S2B). These results indicate that ZFP36L1 and ZFP36L2 predominantly interact with the UAUUUAUU sequence of the ARE1 region of the LDLR mRNA 3′-UTR.

### ZFP36L1 and ZFP36L2 destabilize LDLR mRNA

**Figure 3.**
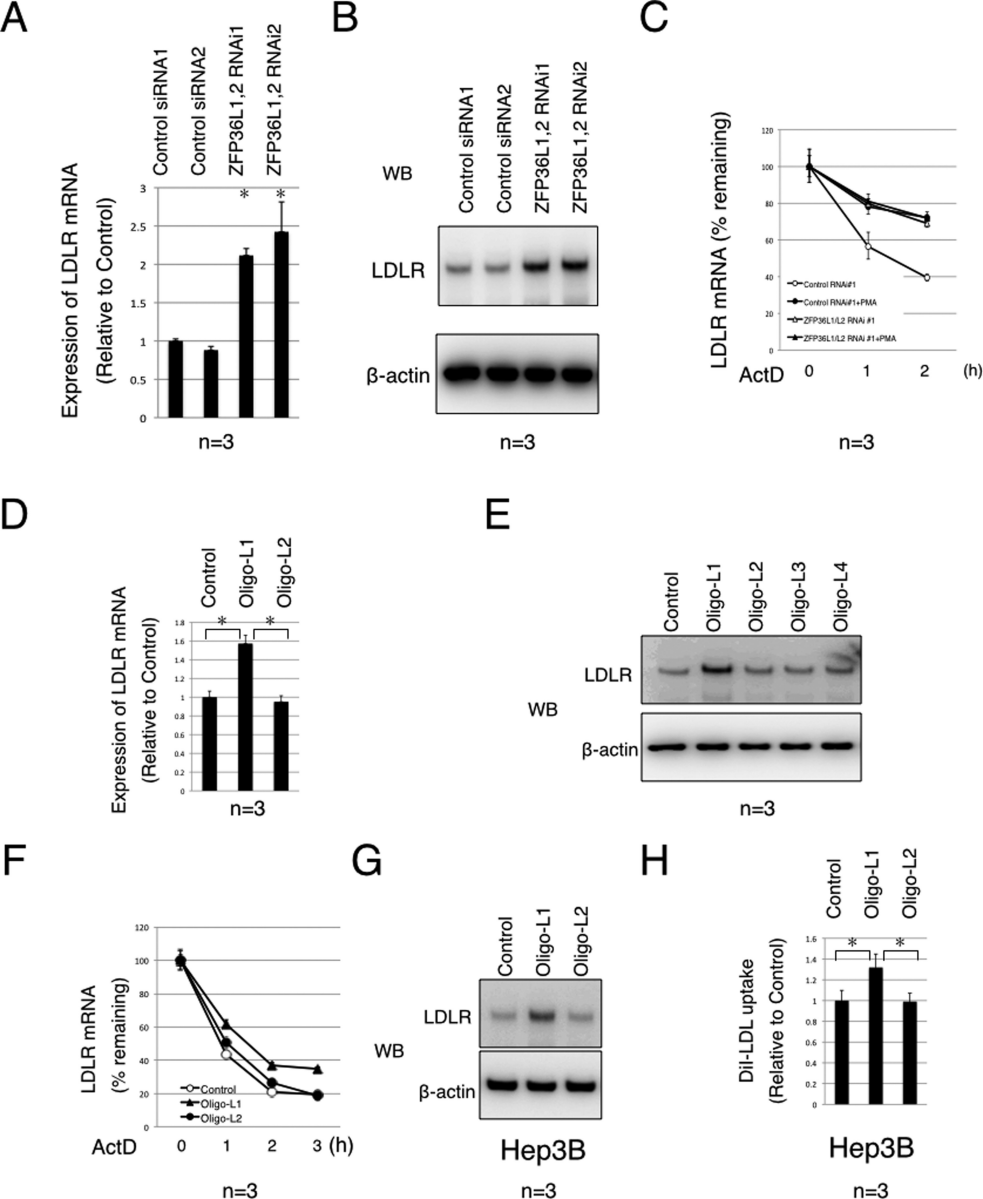
LDLR mRNA is destabilized by ZFP36L1 and ZFP36L2. (**A**) HeLa cells were transfected with ZFP36L1 and ZFP36L2 siRNAs. Forty-eight hours after transfection, cells were harvested, total RNA was extracted and quantitative RT-PCR (qPCR) was performed using primers specific to LDLR mRNA and β-actin mRNA. Results were normalized to β-actin mRNA levels. Error bars show standard deviation of the mean. *P*-values against control were calculated using Student's *t*-test.*P < 0.002; *n* = 3 for each group. (**B**) Forty-eight hours after transfection, cells were harvested and the lysates were subjected to western blot analysis using the indicated antibodies. (**C**) Forty-eight hours after transfection, cells were treated with ActD and chased for the indicated time with or without PMA (PMA treatment commenced 10 min after ActD treatment). Total RNA was extracted and qPCR was performed using primers specific to LDLR mRNA and β-actin mRNA. Results were normalized to the levels of β-actin mRNA. Error bars show standard deviation of the mean. (**D**) HeLa cells were transfected with the indicated oligos. Twenty-four hours after transfection, cells were harvested and total RNA was extracted. Quantitative RT-PCR (qPCR) was performed using primers specific for LDLR and β-actin. Results were normalized to β-actin mRNA levels. Error bars show standard deviation of the mean. *P*-values against control were calculated using Student's *t*-test. *P < 0.002; *n* = 3 for each group. (**E**) HeLa cells were transfected with the indicated oligos. Twenty-four hours after transfection, cells were harvested and the lysates were subjected to western blot analysis using the indicated antibodies. (**F**) HeLa cells were transfected with the indicated oligos. Twenty-four hours after transfection, cells were treated with ActD and chased for the indicated times. Total RNA was extracted and qPCR was performed using primers specific to LDLR mRNA and β-actin mRNA. Results were normalized to the levels of β-actin mRNA. Error bars show standard deviation of the mean. (**G**) Hep3B cells were transfected with the indicated oligos. Twenty-four hours after transfection, cells were harvested and the lysates were subjected to western blot analysis using the indicated antibodies. (**H**) Hep3B cells were transfected with the indicated oligos. Twenty-four hours after transfection, cells were treated with DiI-LDL for 1 h. The cells were then lysed in RIPA buffer and the ratio of DiI-LDL fluorescence/protein concentration was measured. Error bars show standard deviation of the mean. *P*-values against control were calculated using Student's *t*-test. *P < 0.002; *n* = 3 for each group. The data are representative of at least three independent experiments. The number below the figure indicates the number of times we replicated the experiment. Data from one of the independent experiments are shown in Supplementary Figure S4A–H.

To investigate the significance of the interaction between LDLR mRNA and ZFP36L1 or ZFP36L2, we performed double siRNA-mediated knockdown of ZFP36L1 and ZFP36L2. We used HeLa cells because siRNA-mediated gene silencing is more efficient in HeLa cells than in HEK293T cells (15). We first examined the efficiency of knockdown using qPCR (Supplementary Figure S3A). We then examined the effect of this knockdown on LDLR mRNA and protein levels. We found that knockdown of ZFP36L1 and ZFP36L2 together resulted in an increase in both LDLR mRNA and protein (Figure 3A and B, Supplementary Figure S3B). Next, we examined the effect of siRNA on LDLR mRNA stability using an ActD chase experiment. We found that LDLR mRNA in cells transfected with ZFP36L1 and ZFP36L2 siRNA was clearly more stabilized than that in control siRNA-transfected cells (Figure 3C, Supplementary Figure S3C). We also observed PMA-mediated LDLR mRNA stabilization in cells transfected with control siRNA but, interestingly, not in cells transfected with ZFP36L1 and ZFP36L2 siRNAs (Figure 3C, Supplementary Figure S3C). These results suggest that ZFP36L1 and ZFP36L2 are LDLR mRNA-destabilizing factors that are indispensable for PMA-mediated stabilization of LDLR mRNA. ZFP36L1 and ZFP36L2-mediated LDLR mRNA destabilization was also observed in other cell lines, including 293T cells and Hep3B cells, indicating that this regulation is conserved among these cells (Supplementary Figure S3D).

To further confirm that the ZFP36L1 and ZFP36L2-mediated destabilization of LDLR mRNA is caused by direct interaction, we examined the effect of LNA-modified oligonucleotides (as used in the experiment presented in Figure 2D) on the levels of LDLR mRNA and LDLR protein. We transfected these oligonucleotides into HeLa cells and found that Oligo-L1 increased the levels of LDLR mRNA and protein (Figure 3D and E, Supplementary Figure S3E). In contrast, Oligo-L2, -L3 and -L4 had no effect on the levels of LDLR mRNA or protein (Figure 3D and E, Supplementary Figure S3E). In addition, as expected, Oligo-L1 had no effect on the levels of VEGFA or PLK3 mRNAs (Supplementary Figure S3F). We also examined the effect of Oligo-L1 on the stability of LDLR mRNA in HeLa and 293T cells using an ActD chase experiment and we found that Oligo-L1 stabilized LDLR mRNA in these cell lines (Figure 3F, Supplementary Figure S3G and H). We then used two further oligonucleotides, Oligo-L5 and Oligo-L6. Oligo-L5 is a point mutant of Oligo-L1 (T8G) and Oligo-L6 is a scrambled oligonucleotide of Oligo-L1. Neither of these oligonucleotides could inhibit the interaction between LDLR mRNA and ZFP36L1, nor could they increase the stability of LDLR mRNA or the levels of LDLR protein in cells (Supplementary Figure S3I and J)

We finally used human liver-derived Hep3B cells, because we anticipate that this approach could potentially increase the levels of LDLR protein in the liver, thereby lowering blood LDL cholesterol levels. We transfected Oligo-L1 and Oligo-L2 into Hep3B cells and again found that Oligo-L1 stabilized LDLR mRNA and increased the levels of LDLR protein (Figure 3G, Supplementary Figure S3K and L). We then examined the effect of these oligonucleotides on LDL incorporation in Hep3B cells using DiI-labeled LDL. As expected, we found that Oligo-L1 increased LDL incorporation into Hep3B cells (Figure 3H). These results indicate that, LNA-modified antisense oligonucleotides can increase the LDL-uptake activity of liver-derived cells.

### ZFP36L1 is regulated by phosphorylation downstream of ERK

**Figure 4.**
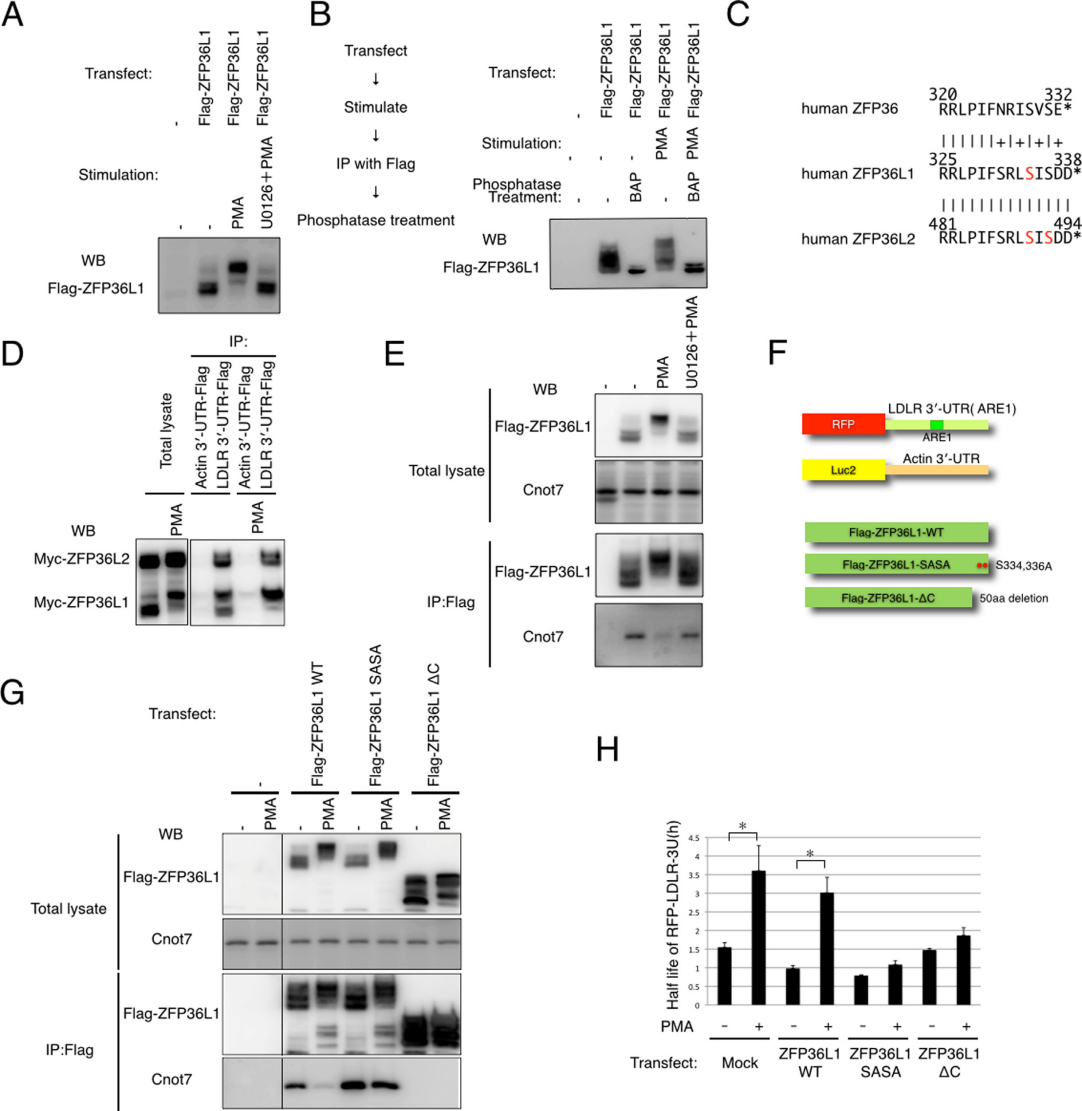
ERK pathway phosphorylates and inhibits ZFP36L2. (**A**) 293T cells were transfected with Flag-tagged ZFP36L1. Twenty-four hours after transfection, cells were treated with PMA (for 30 min) and U0126 (for 45 min), as indicated. Cell lysates were subjected to western blot analysis using anti-Flag antibody. (**B**) 293T cells were transfected with Flag-tagged ZFP36L1. Cells were treated with or without PMA (for 30 min) and the lysates were subjected to IP. Immunopurified Flag-ZFP36L1 was treated with or without bacterial acid phosphatase (BAP) and samples were subjected to western blot analysis using anti-Flag antibody. (**C**) Alignment of C-terminal amino acid sequences in the human ZFP36 family. The C-terminal end of each protein is shown as (*). Identical residues are shown by (|) and similar residues are shown by (+). The serine residues identified as being phosphorylated downstream of ERK are shown in red. (**D**) 293T cells were transfected with Myc-tagged ZFP36L1 or ZFP36L2 and then treated with or without PMA (for 30 min). Cell lysates were subjected to IP using the Flag-antibody and the indicated bait RNAs. Co-immunoprecipitated proteins were subjected to western blot analysis using anti-MYC (9E10) antibody. (**E**) 293T cells were transfected with Flag-tagged ZFP36L1, then treated with PMA (for 30 min) and U0126 (for 45 min) as indicated, and lysates were subjected to IP. Co-immunoprecipitated proteins were subjected to western blot analysis using the indicated antibodies. (**F**) Schematic representation of the constructs used in this experiment. (**G**) 293T cells were transfected with the indicated constructs. Twenty-four hours after transfection, cells were treated with or without PMA (for 30 min), and lysates were subjected to IP. Co-immunoprecipitated proteins were subjected to western blot analysis using the indicated antibodies. (**H**) HeLa cells were transfected with RFP-LDLR 3′-UTR (ARE1) or Luc2-β-Actin-UTR along with the indicated ZFP36L1 constructs. Twenty-four hours after transfection, cells were treated with ActD and PMA (PMA treatment commenced 15 min after ActD treatment), as indicated. Two hours after ActD treatment, cells were harvested and total RNA was extracted. Quantitative RT-PCR (qPCR) was performed using RFP and Luc2-specific primers. Results were normalized to the levels of Luc2 mRNA. The data are representative of at least three independent experiments.

Next, we investigated the underlying mechanisms of PMA-ERK-mediated LDLR mRNA stabilization. Given that ERK is a critical kinase in PMA-mediated LDLR mRNA stabilization (4), we examined whether ZFP36L1 is phosphorylated downstream of ERK. We found that PMA treatment induced an electrophoretic mobility shift of ZFP36L1, which could be reversed when cells were treated with PMA and U0126, a specific inhibitor of the ERK pathway (Figure 4A). We also found that the mobility shift of Flag-ZFP36L1, which we immunopurified from Flag-ZFP36L1-overexpressing and PMA-treated 293T cells, could be reversed by treatment with bacterial alkaline phosphatase (Figure 4B). These results indicate that ZFP36L1 is phosphorylated downstream of ERK. We then analyzed the ERK-dependent phosphorylation sites using an iTRAQ-based quantitative MS approach. We immunopurified Flag-ZFP36L1 protein from mock-, PMA- or PMA + U0126-treated 293T cells and determined the ERK-dependent phosphorylation sites. We found that phosphorylation of the C-terminal serine-334 residue of ZFP36L1 and of the C-terminal serine-493 and -495 residues of ZFP36L2 was increased upon PMA treatment, but was reversed by U0126 treatment (Figure 4C, Supplementary Figure S5A). This result indicates that the phosphorylation of these residues is ERK -dependent. We also analyzed the phosphorylation of endogenous ZFP36L1, which we purified from 293T cell lysate using a Flag-tagged LDLR ARE1 region (Supplementary Table S1), and found that the C-terminal serine-334 residue of endogenous ZFP36L1 is also phosphorylated upon PMA treatment (Supplementary Figure S5B and C).

To understand the function of ZFP36L1 phosphorylation, we first examined whether PMA treatment decreases the RNA-binding ability of ZFP36L1 and ZFP36L2. We found that PMA treatment slightly increased the interaction between Myc-ZFP36L1 and LDLR mRNA, while that with Myc-ZFP36L2 was not affected by PMA treatment (Figure 4C). We found that the RNA-binding ability of endogenous ZFP36L1 was also slightly increased by PMA treatment (Supplementary Figure S5B). These results indicate that PMA treatment does not inhibit the RNA-binding ability of ZFP36L1 and ZFP36L2.

The CCR4-NOT deadenylase complex has recently been shown to interact with the C-terminus of ZFP36 (16,17); therefore, we next examined whether ZFP36L1 interacts with the CCR4-NOT deadenylase complex and whether this interaction is affected by PMA. We found that CNOT7, a critical enzymatic component of CCR4-NOT deadenylase, interacts with ZFP36L1 and interestingly, this interaction is inhibited by PMA treatment and reversed by U0126 treatment, indicating that this regulation is mediated by the ERK pathway (Figure 4E). To confirm that this effect was due to C-terminal phosphorylation, we constructed the ZFP36L1-ΔC mutant (in which C-terminal amino acid residues, including all conserved phosphorylation sites, were deleted) and the ZFP36L1-SASA mutant (in which serine-334 and -336 were mutated to alanine residues, mimicking constitutive de-phosphorylation) (Figure 4F). We then examined the interaction between CNOT7 and these ZFP36L1 mutants and found that the ZFP36L1-SASA mutant retained the ability to interact with CNOT7 after PMA treatment, whereas the ZFP36L1-ΔC mutant did not (Figure 4G). Surprisingly, we also found that PMA treatment still caused an electrophoretic mobility shift of the ZFP36L1-SASA mutant. These results indicate that the interaction between ZFP36L1 and CNOT7 is regulated by ERK-mediated C-terminal phosphorylation of ZFP36L1. Furthermore, C-terminal phosphorylation of ZFP36L1 is not responsible for the PMA-mediated electrophoretic mobility shift.

We considered the possibility that ZFP36 and ZFP36L2 could also be regulated by PMA because the C-terminal region is highly conserved in members of the ZFP36 protein family (Figure 4C). We examined the interaction between CNOT7 and ZFP36 or ZFP36L2 and found that ZFP36L2, in addition to ZFP36 and ZFP36L1, also interacts with CNOT7 and that the interactions between CNOT7 and ZFP36 or ZFP36L2 are inhibited by PMA treatment (Supplementary Figure S5E). Thus, ERK-mediated regulation may be conserved among members of the ZFP36 family.

We further validated the function of ZFP36L1 phosphorylation in LDLR mRNA-destabilization. We transfected the RFP-LDLR-3′-UTR expression vector and a luciferase-β-actin-3′-UTR vector along with ZFP36L1-WT, ZFP36L1-SASA or ZFP36L1-ΔC into 293T cells and preformed ActD chase experiments with or without PMA treatment. We observed PMA-mediated stabilization of RFP-LDLR-3′-UTR in mock- and wild-type-ZFP36L1-transfected cells, but not in ZFP36L1-SASA or ZFP36L1-ΔC mutant-transfected cells (Figure 4F and H). These results indicate that ZFP36L1 is regulated by PMA, and that C-terminal phosphorylation of ZFP36L1 is indispensable for ERK-mediated LDLR-mRNA stabilization.

### RSK directly phosphorylates ZFP36L1 downstream of ERK

**Figure 5.**
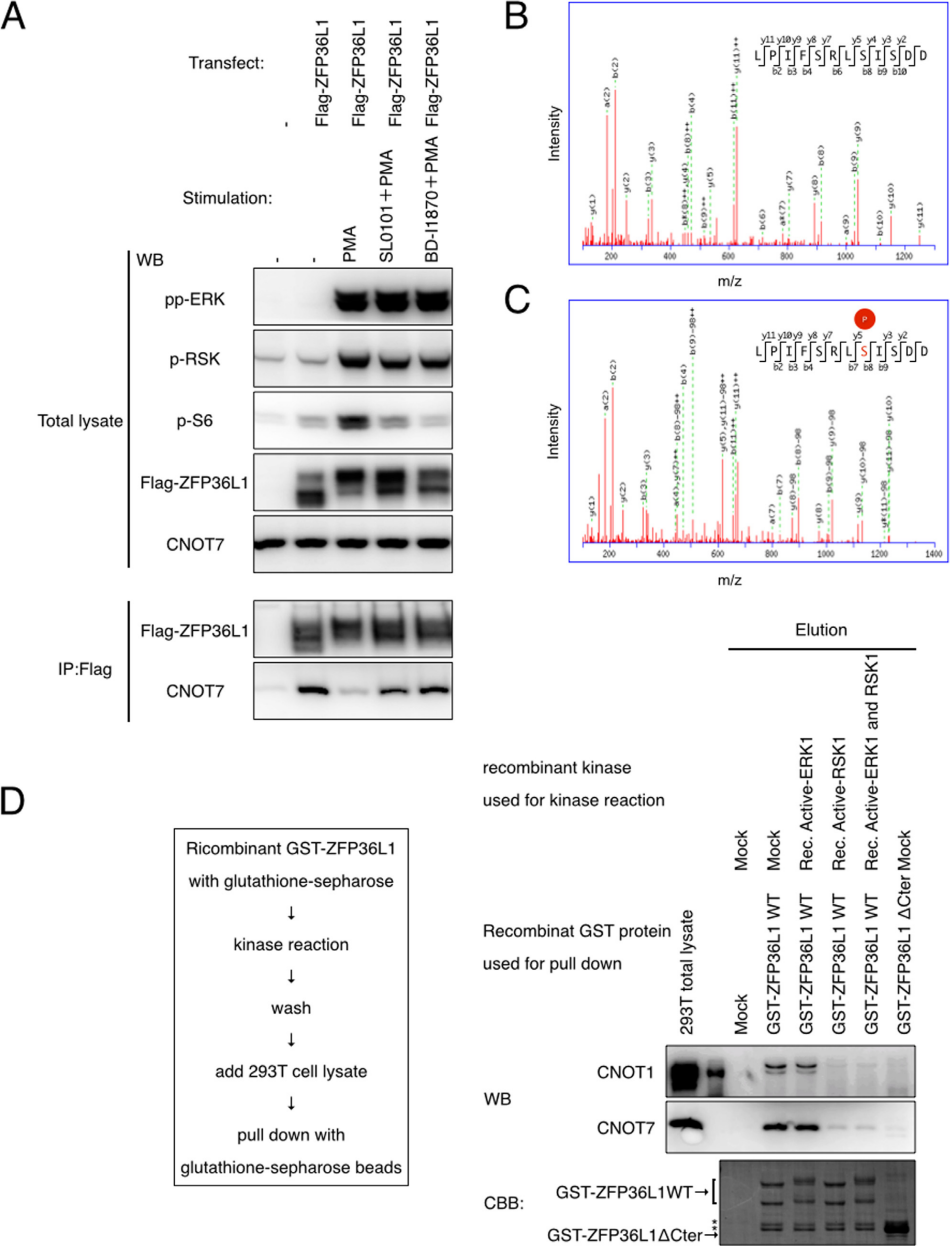
RSK1 directly phosphorylates the C-terminus of ZFP36L1. (**A**) 293T cells were transfected with Flag-tagged ZFP36L1. Twenty-four hours after transfection, cells were treated with PMA (for 30 min) and BI-D1870 or SL0101 (for 45 min) as indicated, and lysates were subjected to IP with the anti-Flag antibody. Co-immunoprecipitated proteins were subjected to western blot analysis using the indicated antibodies. Five percent of the initial amount of cleared 293T lysate was loaded as total lysate. (**B**) GST-ZFP36L1 protein was incubated with or without active recombinant RSK1 under phosphorylation conditions for 30 min. Phosphorylation of GST-ZFP36L1 C-terminal peptide was analyzed by MS. The MS/MS spectrum used to identify the m/z 681.8604-peak as the LPIFSRLSISDD peptide fragment of ZFP36L1 is shown. (**C**) MS/MS spectrum used to identify the m/z 721.8421-peak as the serine-8 phosphorylated LPIFSRLSISDD peptide fragment of ZFP36L1. (**D**) GST-ZFP36L1 protein was incubated with the indicated recombinant kinase under phosphorylation conditions for 30 min, washed, then added to 293T cell lysate and then subjected to pulldown with glutathione-sepharose. Eluted proteins were subjected to western blot analysis using the indicated antibodies and subjected to Coomassie Brilliant Blue (CBB) staining. The asterisk shows a nonspecific band. Five percent of the initial amount of cleared 293T cell lysate was loaded as total lysate. These data are representative of at least three independent experiments.

We showed that the C-terminal serine-334 of ZFP36L1 and the C-terminal serine-493 and -495 of ZFP36L2 are phosphorylated downstream of ERK. However, these sites do not match the consensus MAP kinase recognition motifs (SP or TP), indicating that ERK does not directly phosphorylate ZFP36L1. We then examined the possibility that RSK, a major downstream kinase of ERK, directly phosphorylates the C-terminus of ZFP36L1. We first investigated whether BI-D1870 and SL0101, established RSK inhibitors, can reverse PMA-mediated dissociation between ZFP36L1 and CNOT7 protein. We found that BI-D1870 and SL0101 clearly reversed the effect of PMA (Figure 5A, Supplementary Figure S6A). We then examined whether RSK1 directly phosphorylates the C-terminus of ZFP36L1 using recombinant proteins. We incubated *E. coli*-expressed GST-ZFP36L1 with or without active recombinant RSK1 protein under phosphorylation conditions, and analyzed the C-terminal phosphorylation of GST-ZFP36L1 by MS. We found that the C-terminal serine-334 of ZFP36L1 is phosphorylated only when we incubated GST-ZFP36L1 with active RSK1 (Figure 5B and C, Supplementary Figure S6B). We also examined whether active RSK1 inhibits the ability of ZFP36L1 to interact with CNOT proteins. We incubated GST-ZFP36L1 protein with mock buffer, active recombinant ERK1 and/or active recombinant RSK1 under phosphorylation conditions, washed out residual kinase, added 293T cell lysate and performed pulldowns with glutathione-sepharose. We found that GST-ZFP36L1 looses its ability to interact with CNOT1 and CNOT7 proteins when incubated with active recombinant RSK1 (Figure 5D). These results indicate that RSK1 directly phosphorylates the C-terminus of ZFP36L1 downstream of ERK, and inhibits the mRNA destabilization activity of ZFP36L1.

To further confirm our finding that ZFP36L1 and ZFP36L2 are inhibited by PMA treatment, we examined the effect of PMA on the stability of polo-like kinase 3 (PLK3), VEGFA and cellular myelocytomatosis oncogene (cMYC) mRNAs. PLK3 and VEGFA mRNAs have recently been identified as targets of ZFP36L1 (7,8), whereas cMYC mRNA does not bind to ZFP36L1 or ZFP36L2. We found that PLK3 and VEGFA mRNAs were also stabilized by PMA, but that this was not the case for cMYC mRNA (Supplementary Figure S7). These data strongly support the regulation of ZFP36 family proteins by PMA.

## DISCUSSION

We have identified ZFP36L1 and ZFP36L2 as two proteins that specifically interact with LDLR mRNA. We showed that ZFP36L1 and ZFP36L2 destabilize LDLR mRNA and that this activity is inhibited by ERK/RSK1 signaling. Recently, KHSRP, hnRNPD and PTBP1 have been reported to be LDLR-destabilizing proteins (5). However, using our approach, we identified KHSRP as a binding protein for the 3′-UTRs of IFNA1, β-actin and cMYC mRNAs and hnRNPD and PTBP1 were common to all the RNA baits. In spite of the binding of these proteins, IFNA1 and β-actin mRNAs were stable, whereas cMYC mRNA was unstable and was not stabilized by ERK signaling. How these proteins selectively regulate the stability of LDLR mRNA is unclear. It is possible that ZFP36L1 and ZFP36L2 co-ordinately regulate the stability of LDLR mRNA along with KHSRP, hnRNPD and PTBP1.

### Target prediction of ZFP36L1 and ZFP36L2

ZFP36L1 and ZFP36L2 belong to the family of CCCH tandem zinc finger proteins (the ZFP36 family), members of which interact with AREs containing a UAUUUAUU sequence and trigger the degradation of several ARE-containing mRNAs, including PLK3 and VEGFA (7,8,18,19). Consistent with previous reports, we found that ZFP36L1 and ZFP36L2 predominantly interact with the UAUUUAUU sequence of the ARE1 region of the LDLR mRNA, even though the ARE2 region of the LDLR mRNA also contains the same sequence. Interestingly, IFNA1 mRNA, which also has a UAUUUAUU sequence in its 3′-UTR, is stable in cells and ZFP36L1 does not interact strongly with the 3′-UTR of this mRNA (Figure 1B, Supplementary Table S2). Our results suggest that the UAUUUAUU sequence is necessary but not sufficient for ZFP36L1 and ZFP36L2 to interact with and destabilize mRNA. Further investigation is required to predict the target mRNAs of ZFP36L1 and ZFP36L2 *in silico*.

### Methods used to identify the regulator of RNA stability

The methods used for (RBP) purification are generally classified into *in vivo* and *in vitro* categories. The *in vivo* purification approach makes use of antisense oligonucleotides, aptamers or MS2-based purification to identify interactions between RNA and RBPs within cells (13,20–24). Use of these methods has elucidated important information on interactions (22–24). On the other hand, the *in vitro* purification approach makes use of cell lysates to purify and identify proteins interacting with *in vitro* synthesized RNA. It is known that some critical regulators of specific RNAs interact specifically with RNA *in vitro* (25). The *in vitro* purification method has several advantages. First, since experimental mRNA expression in cells is not required, this method is sufficiently flexible to enable a wide variety of cells to be used without concerns for transfection efficiency or mRNA expression levels. Second, the amount of bait RNA to be used can be precisely determined to obtain reproducible results that can be subjected to comparison analysis. Third, it is possible to use the RNA sequence of interest, which reduces the amount of nonspecific protein interactions, e.g. with ribosomal proteins or PABPs. Given these advantages, plus our fully automated robotic IP and protein identification system, which is highly sensitive and reproducible (11), we opted to use the *in vitro* purification approach, combined with comparison analysis. This allowed us to demonstrate that ZFP36L1 and ZFP36L2 specifically interact with LDLR mRNA, leading to its destabilization. We also identified several known interactions with other bait RNAs including 7SK-CDK9 and 7SK-LARP7. Thus, our results demonstrate that our *in vitro* purification approach is useful for identifying critical regulators of RNAs, which may be used in the identification of important regulators of other types of RNA.

### Oligonucleotide-based functional validation

We used RNAi-based knockdown to assess the function of ZRP36L1 and ZFP36L2, but as with many RNA-regulating proteins, ZFP36L1 and ZFP36L2 regulate multiple target mRNAs (6). This makes it difficult to determine whether the result of knockdown is directly caused by inhibition of the interaction between the mRNA and the putative regulator, or is a secondary effect. We therefore performed targeted disruption of the interaction between LDLR-mRNA and ZFP36L1 and ZFP36L2 proteins, using LNA- (9) modified antisense oligonucleotides. LNA is a new-generation artificial nucleotide involving a 2′- and 4′-linked ribose moiety. LNA oligonucleotides have several useful characteristics (26): (i) LNA oligonucleotides are resistant to exo- and endonucleases; (ii) LNA oligonucleotides do not evoke RNase H activity when paired with a complementary RNA strand; (iii) LNA oligonucleotides have very high specificity for complementary RNA and can discriminate even a single-base difference; therefore, short oligonucleotides can be used. Thus, we used LNA-modified antisense oligonucleotides to disrupt the interaction between LDLR and ZFP36L1 and ZFP36L2.

A crucial factor in using antisense-oligonucleotides for such a purpose is to show that the effect of the antisense-oligonucleotide is caused by the interaction between the antisense-oligonucleotide and the intended target mRNA. We have shown several lines of evidence to confirm that the effect of the antisense-oligonucleotide (Oligo-L1) was caused by disrupting the interaction between LDLR mRNA and ZFP36L1 and ZFP36L2: (i) Oligo-L1 could inhibit the interaction between ZFP36L1 and LRLD mRNA, without affecting the interaction between LDLR and hnRNPD, hnRNPI or KHSRP, or the interaction of ZFP36L1 with the 3′-UTRs of PLK3 or VEGFA mRNAs (Supplementary Figure S2B). (ii) Oligo-L1 could stabilize LDLR mRNA in cells (Figure 3F, Supplementary Figure S3G, H and K). (iii) Oligo-L1 could increase the level of LDLR mRNA in cells (Figure 3D). (iv) Oligo-L1 could increase the level of LDLR protein in cells (Figure 3E and G, Supplementary Figure S3E and L). (v) Four control oligonucleotides, Oligo-L2, -L3, -L4, -L5 and -L6, could not inhibit the interaction between LDLR mRNA and ZFP36L1, and also could not increase the levels of LDLR mRNA or protein in cells (Figure 3D, E and G, Supplementary Figure S3E, J and L). These results indicate that the effect of Oligo-L1 is caused by disruption of the interaction between LDLR mRNA and ZFP36L1 and ZFP36L2. These results indicate that LNA-modified antisense oligonucleotides are a powerful tool for validating the function of RBPs. As Cibois *et al*. have also reported (27), antisense oligonucleotides allow us to elucidate the binding sites and the function of RNA/RBP interactions in cells.

In addition to validating the function of the interaction, we were able to increase the amount of LDLR protein in cells. In the liver, the LDLR binds to LDL, thereby lowering blood LDL levels. It has been reported that increasing the amount of LDLR protein in the liver could be a therapeutic approach for hyperlipidemia (28). Recently, some reports have shown that an anti-microRNA antisense oligonucleotide efficiently blocked the function of the microRNA, thereby alleviating diseases in rodents and nonhuman primates (29,30). These results demonstrate the potency of antisense oligonucleotide-based therapeutics. If appropriate delivery of Oligo-L1 to the liver could be achieved, this could result in a candidate drug for the treatment of hyperlipidemia. We believe that Oligo-L1 can be further developed to treat hyperlipidemia. However, it is also very important to consider off-target effects of antisense oligonucleotides, especially with regard to therapeutic applications.

### Regulation of ZFP36L1

The CCR4-NOT poly (A) deadenylase complex is composed of more than 10 proteins, including CNOT7. It is known to interact with ZFP36 and ZFP36L1 and to act as an important effector complex of ZFP36-mediated mRNA-destabilization (16,17). We show in this report that PMA treatment causes dissociation of ZFP36L1 and CNOT7, and does not cause the dissociation of LDLR and ZFP36L1/L2 (Figure 4D). In the case of ZFP36L1, the interaction is somewhat increased by PMA treatment (Figure 4D, Supplementary Figure S5B); however, the mechanisms and meaning of enhanced PMA-mediated binding of ZFP36L1 to LDLR mRNA are still unknown. Recently, MAPKAPK2, a downstream kinase of p38, has been shown to phosphorylate ZFP36, but not in the C-terminal region, leading to dissociation of ZFP36 and CNOT7 (16,17). These findings indicate that ZFP36 family proteins are regulated by at least two independent signaling pathways. It is an open question, whether these pathways commonly regulate all ZFP36 family proteins or whether there are differences in regulation between ZFP36 family proteins. It will be important to investigate in detail the mechanisms of these regulatory pathways and to clarify their functional consequences.

## SUPPLEMENTARY DATA

Supplementary Data are available at NAR Online.

SUPPLEMENTARY DATA
