# Supplementary material for: ZFP36L1 and ZFP36L2 control LDLR mRNA stability via the ERK–RSK pathway
Source: Nucleic Acids Res. 2014 Aug 8;42(15):10037–49. doi: 10.1093/nar/gku652 (PMC4150769; doi:10.1093/nar/gku652)
Supplement: SUPPLEMENTARY DATA [file supp_42_15_10037__index.html]

ZFP36L1 and ZFP36L2 control LDLR mRNA stability via the ERK–RSK pathway — ZFP36L1 and ZFP36L2 control LDLR mRNA stability via the ERK–RSK pathway — SUPPLEMENTARY DATA 

# ZFP36L1 and ZFP36L2 control LDLR mRNA stability via the ERK–RSK pathway

## SUPPLEMENTARY DATA

**Files in this Data Supplement:**

- SUPPLEMENTARY DATA
- SUPPLEMENTARY DATA
