# Supplementary material for: ZFP36L1 and ZFP36L2 control LDLR mRNA stability via the ERK–RSK pathway
Source: Nucleic Acids Res. 2014 Aug 8;42(15):10037–49. doi: 10.1093/nar/gku652 (PMC4150769; doi:10.1093/nar/gku652)

Supplemental Information

(a) Title

ZFP36L1 and ZFP36L2 control LDLR mRNA stability via the ERK-RSK pathway

(b) Authors

Shungo Adachi[1,2], Masae Homoto[1], Rikou Tanaka[1,2], Yusaku Hioki[1], Hiroshi Murakami[3], Hiroaki Suga[4], Masaki Matsumoto[5], Keiichi I. Nakayama[5], Tomohisa Hatta[1], Shun-ichiro Iemura[1,6], Thoru Natsume[1]

Inventory of Supplemental Information

1, Supplemental Table Legend (1-4)

2, Supplemental Figure Legend (1-7)

3, Supplemental Tables (1-4)

4, Supplemental Figures (1-7)

1, Supplemental Table Legend

Supplementary Table S1

Sequence of cDNAs used to prepare RNA baits.

Supplementary Table S2

List of all MS-identified proteins when several RNAs were used as bait.

Supplementary Table S3

List of proteins identified to specifically interact with LDLR-3′-UTR-Flag.

Supplementary Table S4

List of proteins identified to specifically interact with 7SK-RNA-Flag.

2, Supplemental Figure Legend

Supplementary Figure S1 Flag-conjugation of bait RNA and validation of RNA-Flag based co-immunoprecipitation

(A) Overview of hydrazide-mediated Flag-conjugation to the 3′-terminal of bait RNA.

(B) The Flag-conjugated 21-basepair sense strand RNA and unconjugated RNA were resolved on a Urea-PAGE gel and visualized by ethidium bromide staining.

(C) Overview of Flag-based RNA co-immunoprecipitation experiment.

(D) 293T cells were transfected with HA-tagged MS2 and were then subjected to immunoprecipitation (IP) with anti-Flag antibody and 10 pmol of the indicated bait RNA. Co-immunoprecipitated RNA and proteins eluted using the Flag-peptide were subjected to western blot analysis using anti-HA antibody. Five percent of the initial amount of cleared 293T lysate was loaded as total lysate (TL).

Supplementary Figure S2 Antisense-oligonucleotide-mediated Disruption of the interaction between RNA and RBPs

(A) Schematic representation of the 3′-UTR of LDLR mRNA and location of the designed oligos. ARE 1 is indicated by red boxes, and designed oligos are indicated by blue lines.

(B) 293T cells were lysed with lysis buffer and subjected to immunoprecipitation using the indicated combinations of oligos and bait RNAs. Immunoprecipitated proteins were subjected to western blot analysis with the indicated antibodies and immunoprecipitated RNA was visualized with ethidium bromide staining.

Supplementary Figure S3 ZFP36L1 and ZFP36L2 mediated destabilization of LDLR mRNA

(A) 293T cells, Hep3B and HeLa cells were transfected with the indicated siRNAs. Forty-eight hours after transfection, cells were harvested, total RNA was extracted and quantitative RT-PCR (qPCR) was performed using primers specific to human ZFP36L1, human ZFP36L2 and human beta-actin. Results were normalized to beta-actin mRNA.

(B) Forty-eight hours after transfection, cells were harvested and the lysates were subjected to western blot analysis. Intensity of individual LDLR and beta-actin bands was quantified using Multi Gauge software (Fuji Photo Film Co., Ltd). Results were normalized to beta-actin. Error bars show standard deviation of the mean. P-values were calculated using Student’s t-test. *$P<0.001$; $n=3$ for each group.

(C) Half-lives of LDLR mRNA were calculated by fitting an exponential decay curve to all time points of 3 independent experiments. Figures in the graph indicate the mean value of the mRNA decay half-lives. Error bars show standard deviation of the mean. P-values were calculated using Student’s t-test. *$P<0.001$; $n=3$ for each group.

(D) Half-lives of LDLR mRNA were calculated as above. Figures in the graph indicate the mean value of the mRNA decay half-lives. Error bars show standard deviation of the mean. P-values were calculated using Student’s t-test.**$P<0.01$; $n=3$ for each group.

(E) HeLa cells were transfected with the indicated oligos. Twenty-four hours after transfection, cells were harvested and the lysates were subjected to western blot analysis. Intensity of individual bands was quantified using Multi Gauge software (Fuji Photo Film Co., Ltd). Results were normalized to beta-actin. P-values were calculated using Student's t-test.****P<0.05; n=3 for each group.

(F) HeLa cells were transfected with the indicated oligos. Twenty-four hours after transfection, cells were harvested, total RNA was extracted and quantitative RT-PCR (qPCR) was performed using primers specific to human VEGFA, human PLK3 and human beta-actin. Results were normalized to beta-actin mRNA.

(G) HeLa cells were transfected with the indicated oligos. Half-lives of LDLR mRNA were calculated as above. Figures in the graph indicate the mean value of the mRNA decay half-lives.. P-values were calculated using Student's t-test.*P<0.001; n=3 for each group.

(H) 293T cells were transfected with the indicated oligos. Half-lives of LDLR mRNA were calculated as above. Figures in the graph indicate the mean value of the mRNA decay half-lives. P-values were calculated using Student's t-test.***P<0.02; n=3 for each group.

(I) 293T cells were lysed with lysis buffer and the cleared lysates were subjected to immunoprecipitation with the indicated LDLR 3′-UTR bait RNAs and the indicated oligos. Immunoprecipitated proteins were subjected to western blot analysis with the anti-ZFP36L1 antibody.

(J) HeLa cells were transfected with the indicated oligos. Twenty-four hours after transfection, cells were harvested and the lysates were subjected to western blot analysis using the indicated antibodies.

(K) Hep3B cells were transfected with the indicated oligos. Half-lives of LDLR mRNA were calculated as above. Figures in the graph indicate the mean value of the mRNA decay half-lives. P-values were calculated using Student's t-test.*P<0.001; n=3 for each group.

(L) HeLa cells were transfected with the indicated oligos. Twenty-four hours after transfection, cells were harvested and the lysates were subjected to western blot analysis. Intensity of individual bands was quantified as above. P-values were calculated using Student's t-test.**$P<0.01$; $n=3$ for each group.

Supplementary Figure S4 One of the independent sets of data from Figure 3

(A) Data from one of the independent experiments of Figure 3A. P-values were calculated using Student's t-test.*$P<0.001$; $n=3$ for each group.

(B) Data from one of the independent experiments of Figure 3B.

(C) Data from one of the independent experiments of Figure 3C.

(D) Data from one of the independent experiments of Figure 3D. P-values were calculated using Student's t-test.*$P<0.001$; $n=3$ for each group.

(E) Data from one of the independent experiments of Figure 3E.

(F) Data from one of the independent experiments of Figure 3F.

(G) Data from one of the independent experiments of Figure 3G.

(H) Data from one of the independent experiments of Figure 3H. P-values were calculated using Student's t-test.***$P<0.02$; $n=3$ for each group.

Supplementary Figure S5 Regulation of ZFP36 family proteins downstream of ERK pathway

(A) Phosphorylated ZFP36L1 and ZFP36L2 peptides were identified and quantified using iTRAQ-based MS/MS analysis. Three peptides, which are used to quantity the increase in phosphorylation after PMA treatment and the reduction in phosphorylation after U0126 treatment, are shown. The phosphorylated serine residue is coloured red. The quantity of phosphorylated

peptide was normalized to the mock treated control sample. P-values were calculated using Student's t-test. ****P<0.05; n=3 for each group.

(B) 293T cells were lysed with lysis buffer and subjected to immunoprecipitation using the indicated bait RNAs. Immunoprecipitated proteins were subjected to western blot analysis with anti ZFP36L1 antibody. The amount of immunoprecipitated ZFP36L1 protein was quantified by measuring the intensity of bands. P-values were calculated using Student's t-test. **P<0.01; n=3 for each group.

(C) 293T cells were lysed with lysis buffer and subjected to immunoprecipitation as indicated in figure S5B. Immunoprecipitated proteins were subjected to MS analysis. The quantity of phosphorylated peptide was normalised to common peptide (TFHTIGFCPYGPR) from ZFP36L1 and ZFP36L2. The phosphorylated serine residue is coloured red. P-values were calculated using Student's t-test. *P<0.001; n=3 for each group.

(D) MS/MS spectrum used to identify the phosphorylated peptide fragment of ZFP36L1.

(E) 293T cells were transfected with Flag-tagged ZFP36 and ZFP36L2, as indicated. Twenty four hours after transfection, cells were treated with PMA (for 30 min) and the lysates were subjected to immunoprecipitation with an anti-Flag antibody. Co-immunoprecipitated proteins were subjected to western blot analysis using the indicated antibodies. Five percent of the initial amount of cleared 293T lysate was loaded as total lysate (TL).

Supplementary Figure S6 Ion chromatogram of identified peptides

(A) Quantification of co-immunoprecipitated CNOT7. Co-immunoprecipitated CNOT7 protein was quantified by measuring the intensity of bands. Quantity of co-immunoprecipitated CNOT7 was normalised to immunoprecipitated Flag-tagged ZFP36L1. Error bars show standard deviation of

the mean. P-values were calculated using Student's t-test. *P<0.001; **P<0.01; n=3 for each group.

(B) GST-ZFP36L1 protein was incubated with or without active recombinant RSK1 under phosphorylation conditions for 30 min. Phosphorylation of the C-terminal peptide of GST-ZFP36L1 was analyzed by mass spectrometry. The extracted ion intensities of LPIFSRLSISDD peptide and serine-8-phosphorylated LPIFSRLSISDD peptide were plotted against m/z. The phosphorylated serine residue is coloured red.

Supplementary Figure S7 Stabilization of PLK3 and VEGFA mRNAs by PMA treatment

HeLa cells were treated with actinomycin D (ActD) and chased for the indicated times with or without PMA (HeLa cells were treated with PMA 10 min after the ActD treatment). Total RNA was extracted and used for real-time PCR). (A) Real-time PCR analysis of PLK3 mRNA. (B) Real-time PCR analysis of VEGFA mRNA. (C) Real-time PCR analysis of cMYC mRNA. Results were normalized to beta-actin mRNA.

human 7SK RNA:
ATGTGAGGCGATCTGGCTGCGACATCTGTCACCCCATTGATCGCCAGGGTTGATTCGGCTGATCTGGCTGGCTAGGCGGGTGTCCCCTTCCTCCCTCACCGCTCCATGTGCGTCCCTCCCGAAGCTGCGCGCTCGGTCGAAGAGGACGACCATCCCCGATAGAGGAGGACCGGTCTTCGGTCAAGGGTATACGAGTA
GCTGCGCTCCCCTGCTAGAACCTCCAAACAAGCTCTCAAGGTCCATTTGTAGGAGAACGTAGGGTAGTCAAGCTTCCAAGACTGCAGACACATCCAAATGAGGCGCTGCATGTGGCAGTCTGCCTT

human hnrnpA2/B1-3´ -UTR:
GCTTCTTCCTATTTGCCATGGGTAAGTAGCTTTTGAGTTTTACAATTATTATTATCTTGGGAGACATAGCTGCAGGAGTAAAAGCTTTTTAGGATCATGTTATCTTTCCTTAAAATCTGGTTAGATGGATAATTTCATAACCTATTTTTTTTTTACTCTTTACTTCTGTTGAAACAGGCTTCACTGTATAAATAGGA
GAGGATGAGAGCCCAGAGGTAACAGAACAGCTTCAGGTTATCGAAATAACAATGTTAAGGAAACTCTTATCTCAGTCATGCATAAATATGCAGTGATATGGCAGAAGACACCAGAGCAGATGCAGAGAGCCATTTTGTGAATGGATTGGATTATTTAATAACATTACCTTACTGTGGAGGAAGGATTGTAAAAAAAA
ATGCCTTTGAGACAGTTTCTTAGCTTTTTAATTGTTGTTTCTTTCTAGTGGTCTTTGTAAGAGTGTAGAAGCATTCCTTCTTTGATAATGTTAAATTTGTAAGTTTCAGGTGACATGTGAAACCTTTTTTAAGATTTTTCTCAAAGTTTTGAAAAGCTATTAGCCAGGATCATGGTGTAATAAGACATAACGTTTTT
CCTTTAAAAAAATTTAAGTGCGTGTGTAGAGTTAAGAAGCTGTTGTACATTTATGATT

human MBP3´ -UTR:
AAACCCACCTGGTTCCGGAATCCTGTCCTCAGCTTCTTAATATAACCGCCTTAAAACTTTAATCCCACTTGCCCCTGTTACCTAATTAGAGCAGATGACCCCTCCCCTAATGCCTGCGGAGTTGTGCACGTAGTAGGGTCAGGCCACGGCAGCCTACCGGCAATTTCCGGCCAACAGTTAAATGAGAACATGAAAAC
AGAAAACGGTTAAAACTGTCCCTTTCTGTGTGAAGATCACGTTCCTTCCCCCGCAATGTGCCCCCAGACGCACGTGGGTCTTCAGGGGGCCAGGTGCACAGACGTCCCTCCACGTTCACCCCTCCACCCTTGGACTTTCTTTTCGCCGTGGCTGCGGCACCCTTGCGCTTTTGCTGGTCACTGCCATGGAGGCACAC
AGCTGCAGAGACAGAGAGGACGTGGGCGGCAGAGAGGACTGTTGACATCCAAGCTTCCTTTGTTTTTTTTTCCTGTCCTTCTCTCACCTCCTAAAGTAGACTTCATTTTTCCTAACAGGATTAGACAGTCAAGGAGTGGCTTACTACATGTGGGAGCTTTTGGTATGTGACATGCGGGCTGGGCAGCTGTTAGAGTC
CAACGTGGGGCAGCACAGAGAGGGGGCCACCTCCCCAGGCCGTGGCTGCCCACACACCCCAATTAGCTGAATTCGCGTGTGGCAGAGGGAGGAAAAGGAGGCAAACGTGGGCTGGGCAATGGCCTCACATAGGAAACAGGGTCTTCCTGGAGATTTGGTGATGGAGATGTCAAGCAGGTGGCCTCTGGACGTCACCG
TTGCCCTGCATGGTGGCCCCAGAGCAGCCTCTATGAACAACCTCGTTT

human HIST2H2AA3:
atgtctggtcgtggcaagcaaggaggcaaggcccgcgccaaggccaagtcgcgctcgtcccgcgctggccttcagttcccggtagggcgagtgcatcgcttgctgcgcaaaggcaactacgcggagcgagtgggggccggcgcgcccgtctacatggctgcggtcctcgagtatctgaccgccgagatcctggagct
ggcgggcaacgcggctcgggacaacaagaagacgcgcatcatccctcgtcacctccagctggccatccgcaacgacgaggaactgaacaagctgctgggcaaagtcaccatcgcccagggcggcgtcttgcctaacatccaggccgtactgctccctaagaagacggagagtcaccacaaggcaaagggcaagtgag
gctgacgtccggcccaagtgggcccagcccggcccgcgtctcgaaggggcacctgtgaactcaaaaggctcttttcagagccacccacgttttcaaataaaagagttgttaatgctg

human cMYC-3´ -UTR:
taaggaaaagtaaggaaaacgattccttctaacagaaatgtcctgagcaatcacctatgaacttgtttcaaatgcatgatcaaatgcaacctcacaaccttggctgagtcttgagactgaaagATTTAgccataatgtaaactgcctcaaattggactttgggcataaaagaactttttttatgcttaccatcttttt
tttttctttaacagatttgtATTTAagaattgtttttaaaaaattttaagATTTAcacaatgtttctctgtaaatattgccattaaatgtaaataactttaataaaacgtttatagcagttacacagaatttcaatcctagtatatagtacctagtattataggtactataaaccctaattttttttATTTAagtac
attttgctttttaaagttgatttttttctattgtttttagaaaaaataaaataactggcaaatatatcattgagcc

human Actin 3´ -UTR:
taggcggactatgacttagttgcgttacaccctttcttgacaaaacctaacttgcgcagaaaacaagatgagattggcatggctttatttgtttttttttgttttgttttggttttttttttttttttggcttgactcaggatttaaaaactggaacggtgaaggtgacagcagtcggttggagcgagcatcccccaa
agttcacaatgtggccgaggactttgattgcacattgttgttttttaatagtcattccaaatatgagatgcattgttacaggaagtcccttgccatcctaaaagccaccccacttctctctaaggagaatggcccagtcctctcccaagtccacacaggggaggtgatagcattgctttcgtgtaaattatgtaat
gcaaaatttttttaatcttcgccttaatacttttttattttgttttattttgaatgatgagccttcgtgcccccccttccccctttttgtcccccaacttgagatgtatgaaggcttttggtctccctgggagtgggtggaggcagccagggcttacctgtacactgacttgagacca

human IFNA1-3´ -UTR:
taacatctggtccaacatgaaaacaattcttattgactcatacaccaggtcacgctttcatgaattctgtcatttcaaagactctcacccctgctataactatgaccatgctgataaactgatttatctatttaaatatttatttaactattcataagatttaaattattttgttcatataacgtcatgtgcacct
ttacactgtggttagtgtaataaaacatgttccttatatttactc

human LDLR-3´ -UTR-ARE1-3:
TGATTTTATTTTGACTGATAGTGACCTGTTCGTTGCAACAAATTGATGAGCAATGCTTTTTTATAATGCCAACTTTGTACAAAAAAGCAGGCTTCGAAGGAGATAGAACCACATCTGCCTGGAGTCCCGTCCCTGCCCAGAACCCTTCCTGAGACCTCGCCAGCCTTGTTTTATTCAAAGACAGAGAAGACCAAAGC
ATTGCCTGCCAGAGCTTTGTTTTATATATTTATTCATCTGGGAGGCAGAACAGGCTTCGGACAGTGCCCATGCAATGGCTTGGGTTGGGATTTTGGTTTCTTCCTTTCCTCGTGAAGGATAAGAGAAACAGGCCCGGGGGGACCAGGATGACACCTCCATTTCTCTCCAGGAAGTTTTGAGTTTCTCTCCACCGTGA
CACAATCCTCAAACATGGAAGATGAAAGGGCAGGGGATGTCAGGCCCAGAGAAGCAAGTGGCTTTCAACACACAACAGCAGATGGCACCAACGGGACCCCCTGGCCCTGCCTCATCCACCAATCTCTAAGCCAAACCCCTAAACTCAGGAGTCAACGTGTTTACCTCTTCTATGCAAGCCTTGCTAGACAGCCAGGT
TAGCCTTTGCCCTGTCACCCCCAAATCATGACCCACCCAGTGTCTTTCGAGGTGGGTTTGTACCTTCCTTAAGCCAGGAAAGGGATTCATGGCGTCGGAAATGATCTGGCTGAATCCGTGGTGGCACCGAGACCAAACTCATTCACCAAATGATGCCACTTCCCAGAGGCAGAGCCTGAGTCACCGGTCACCCTTAA
TATTTATTAAGTGCCTGAGACACCCGGTTACCTTGGCCGTGAGGACACGTGGCCTGCACCCAGGTGTGGCTGTCAGGACACCAGCCTGGTGCCCGTCCTCCCGACCCCTACCCACTTCCATTCCCGTGGTCTCCTTGCACTTTCTCAGTTCAGAGTTGTACACTGTGTACATTTGGCATTTGTGTTATTATTTTGCA
CTGTTTTCTGTCGTGTGTGTTGGGATGGGATCCCATATGACCCAGCTTTCTTGTACAAAGTTGGCATTATAAGAAAGCATTGCTTATCAATTTGTTGCAACGAACAGGTCACTATCAGTCAAAATAAAATCATTATTTGCCATCCAGCTGA

human LDLR-3´ -UTR-ARE1-3ΔARE1:
ACCAGGATGACACCTCCATTTCTCTCCAGGAAGTTTTGAGTTTCTCTCCACCGTGACACAATCCTCAAACATGGAAGATGAAAGGGCAGGGGATGTCAGGCCCAGAGAAGCAAGTGGCTTTCAACACACAACAGCAGATGGCACCAACGGGACCCCCTGGCCCTGCCTCATCCACCAATCTCTAAGCCAAACCCCTAA
ACTCAGGAGTCAACGTGTTTACCTCTTCTATGCAAGCCTTGCTAGACAGCCAGGTTAGCCTTTGCCCTGTCACCCCCAAATCATGACCCACCCAGTGTCTTTCGAGGTGGGTTTGTACCTTCCTTAAGCCAGGAAAGGGATTCATGGCGTCGGAAATGATCTGGCTGAATCCGTGGTGGCACCGAGACCAAACTCATT
CACCAAATGATGCCACTTCCCAGAGGCAGAGCCTGAGTCACCGGTCACCCTTAATATTTATTAAGTGCCTGAGACACCCGGTTACCTTGGCCGTGAGGACACGTGGCCTGCACCCAGGTGTGGCTGTCAGGACACCAGCCTGGTGCCCGTCCTCCCGACCCCTACCCACTTCCATTCCCGTGGTCTCCTTGCACTTTC
TCAGTTCAGAGTTGTACACTGTGTACATTTGGCATTTGTGTTATTATTTTGCACTGTTTTCTGTCGTGTGTGTTGGGATGGGATCCCATATGACCCAGCTTTCTTGTACAAAGTTGGCATTATAAGAAAGCATTGCTTATCAATTTGTTGCAACGAACAGGTCACTATCAGTCAAAATAAAATCATTATTTGCCATCC
AGCTGA

human LDLR-3´ -UTR-ARE1-3ΔARE2,3:
TGATTTTATTTTGACTGATAGTGACCTGTTCGTTGCAACAAATTGATGAGCAATGCTTTTTTATAATGCCAACTTTGTACAAAAAAGCAGGCTTCGAAGGAGATAGAACCACATCTGCCTGGAGTCCCGTCCCTGCCCAGAACCCTTCCTGAGACCTCGCCAGCCTTGTTTTATTCAAAGACAGAGAAGACCAAAGC
ATTGCCTGCCAGAGCTTTGTTTTATATATTTATTCATCTGGGAGGCAGAACAGGCTTCGGACAGTGCCCATGCAATGGCTTGGGTTGGGATTTTGGTTTCTTCCTTTCCTCGTGAAGGATAAGAGAAACAGGCCCGGGGGGACCAGGATGACACCTCCATTTCTCTCCAGGAAGTTTTGAGTTTCTCTCCACCGTGA
CACAATCCTCAAACATGGAAGATGAAAGGGCAGGGGATGTCAGGCCCAGAGAAGCAAGTGGCTTTCAACACACAACAGCAGATGGCACCAACGGGACCCCCTGGCCCTGCCTCATCCACCAATCTCTAAGCCAAACCCCTAAACTCAGGAGTCAACGTGTTTACCTCTTCTATGCAAGCCTTGCTAGACAGCCAGGT
TAGCCTTTGCCCTGTCACCCCCAAATCATGACCCACCCAGTGTCTTTCGAGGTGGGTTTGTACCTTCCTTAAGCCAGGAAAGGGATTCATGGCGTCGGAAATGATCTGGCTGAATCCGTGGTGGCACCGAGACCAAACTCATTCACCAAATGATGCCACTTCCCAGAGGCAGAGCCTGAGTCACCGGTC

human LDLR-3´ -UTR-ZFP36L1 binding region:
GATGAATAAATATATAAAACA

TABLE SIII. List of proteins extracted as specificaly interacting with LDLR mRNA-3′-UTR

Symbol [a]	Name [a]
ZFP36L1	butyrate response factor 1
ZFP36L2	butyrate response factor 2

Protein names and Symbols referred to the Entrez Gene database. The proteins identified by a common peptide sequence were indicated ‘or’ in Name column, and ‘l’ in Symbol column.

TABLE SIV. List of proteins extracted as specificaly interacting with 7SK ncRNA

Symbol [a]	Name [a]
BAZ1A	bromodomain adjacent to zinc finger domain protein 1A
CDK9	cell division protein kinase 9
FXR1	fragile X mental retardation syndrome-related protein 1
FXR1lFXR2	fragile X mental retardation syndrome-related protein 1 or 2
GTF3C1	general transcription factor 3C polypeptide 1
GTF3C3	general transcription factor 3C polypeptide 3
GTF3C4	general transcription factor 3C polypeptide 4
GTF3C5	general transcription factor 3C polypeptide 5
HEXIM1	protein HEXIM1
LARP7	la-related protein 7
MEPCE	7SK snRNA methylphosphate capping enzyme
RRP1B	ribosomal RNA processing protein 1 homolog B
SMARCA1lSMARCA5	SWI/SNF related, matrix associated, actin dependent regulator of chromatin, subfamily a, member 1 or 5
SMARCA5	SWI/SNF related, matrix associated, actin dependent regulator of chromatin, subfamily a, member 5

Protein names and Symbols referred to the Entrez Gene database. The proteins identified by a common peptide sequence were indicated ‘or’ in Name column, and ‘l’ in Symbol column.

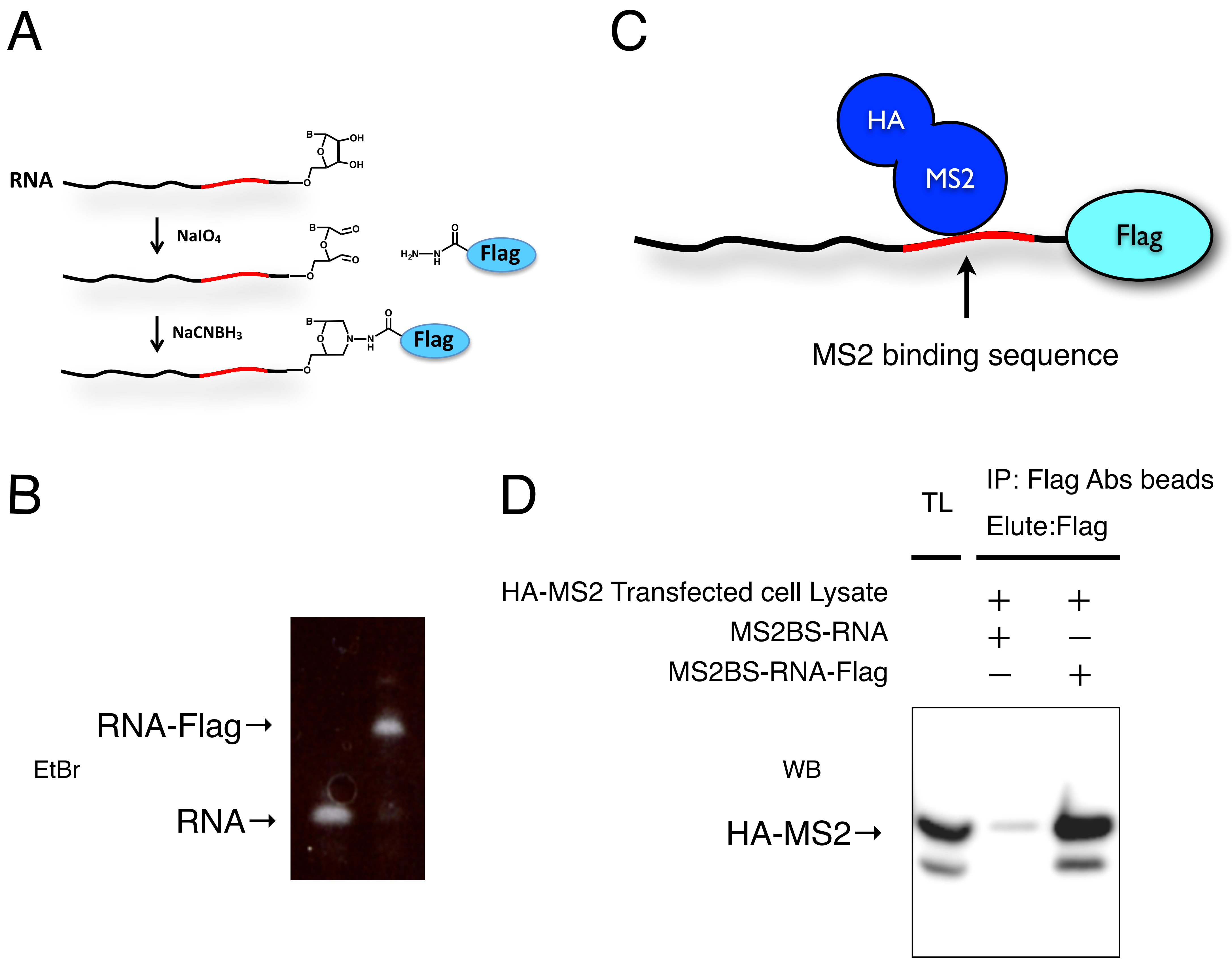
A
RNA
NaIO4
NaCNBH3
Flag
C
HA
MS2
Flag
MS2 binding sequence
B
RNA-Flag→
EtBr
RNA→
D
TL
IP: Flag Abs beads
Elute:Flag
HA-MS2 Transfected cell Lysate + +
MS2BS-RNA + −
MS2BS-RNA-Flag − +
WB
HA-MS2→

Adachi_et_al_FigS2

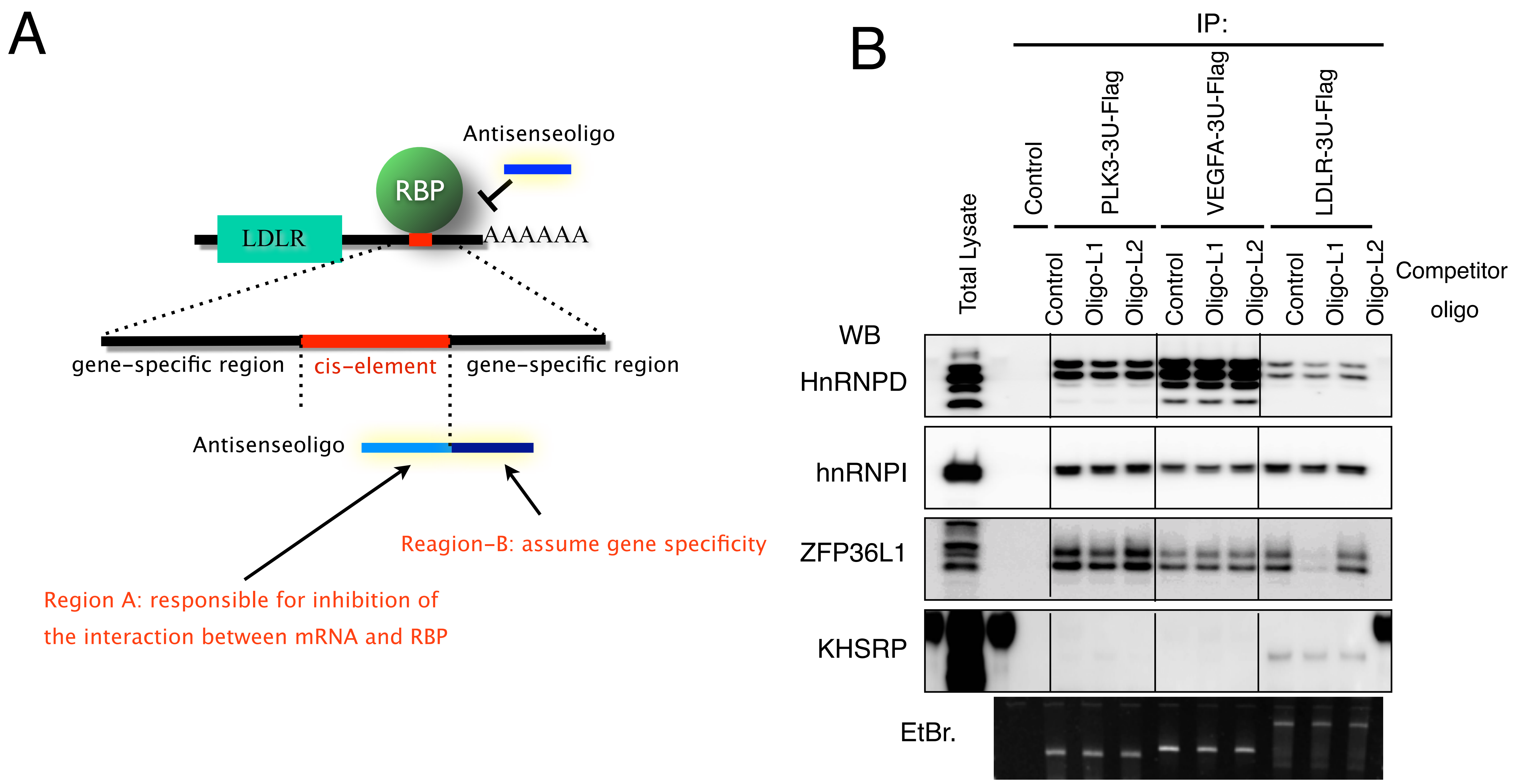

Adachi_et_al_FigS3

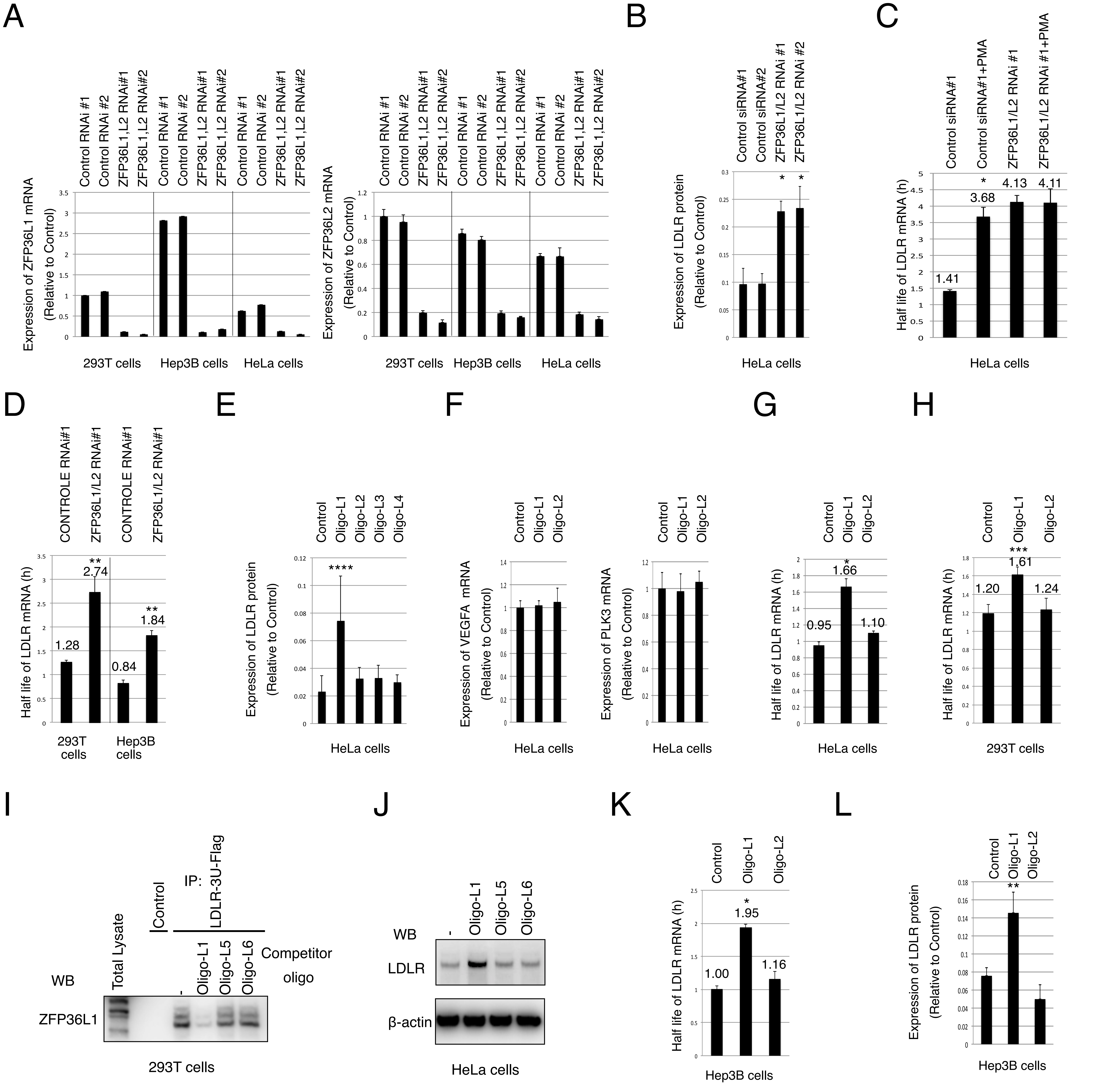

Adachi_et_al_FigS4

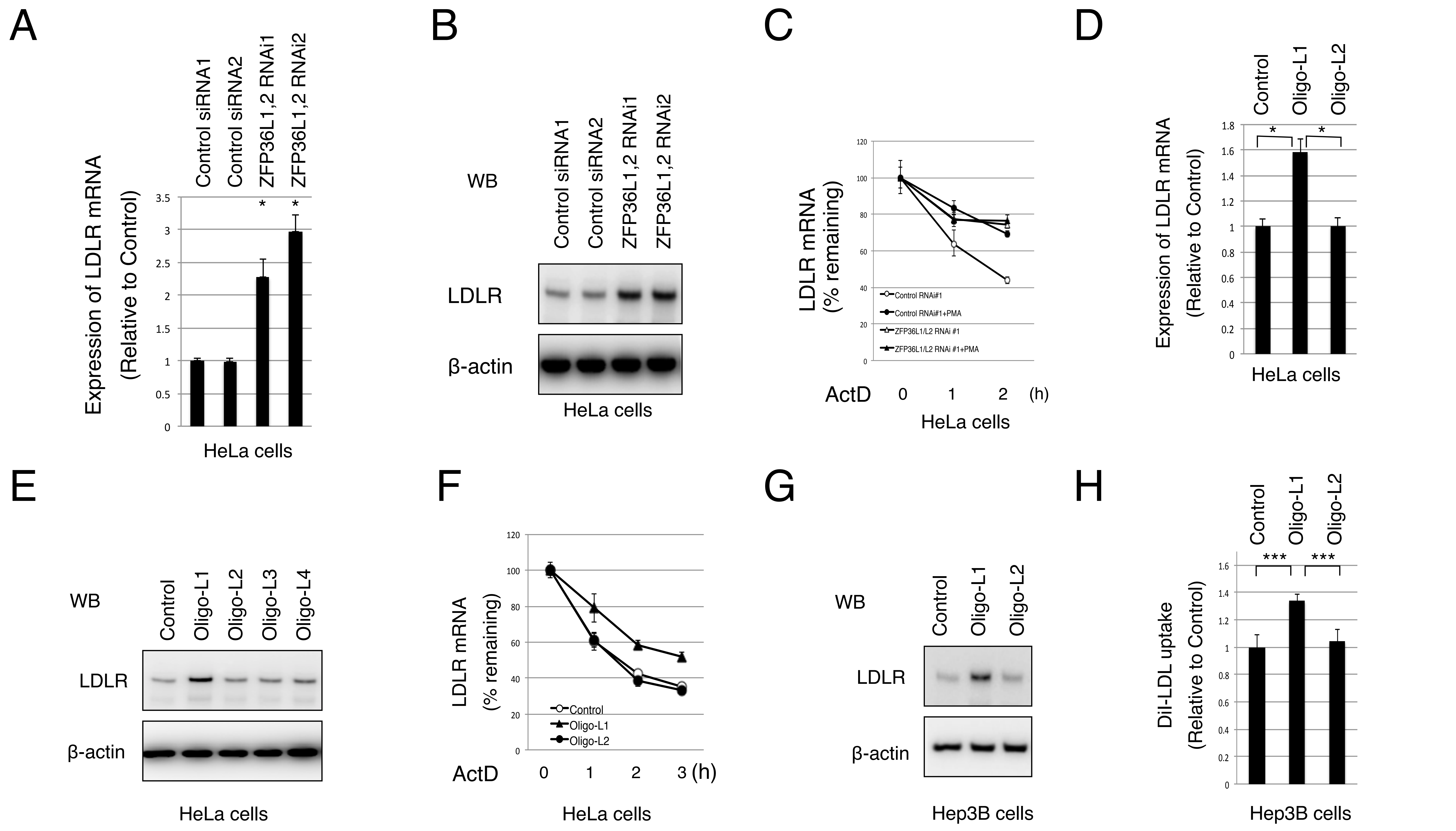

Adachi_et_al_FigS5

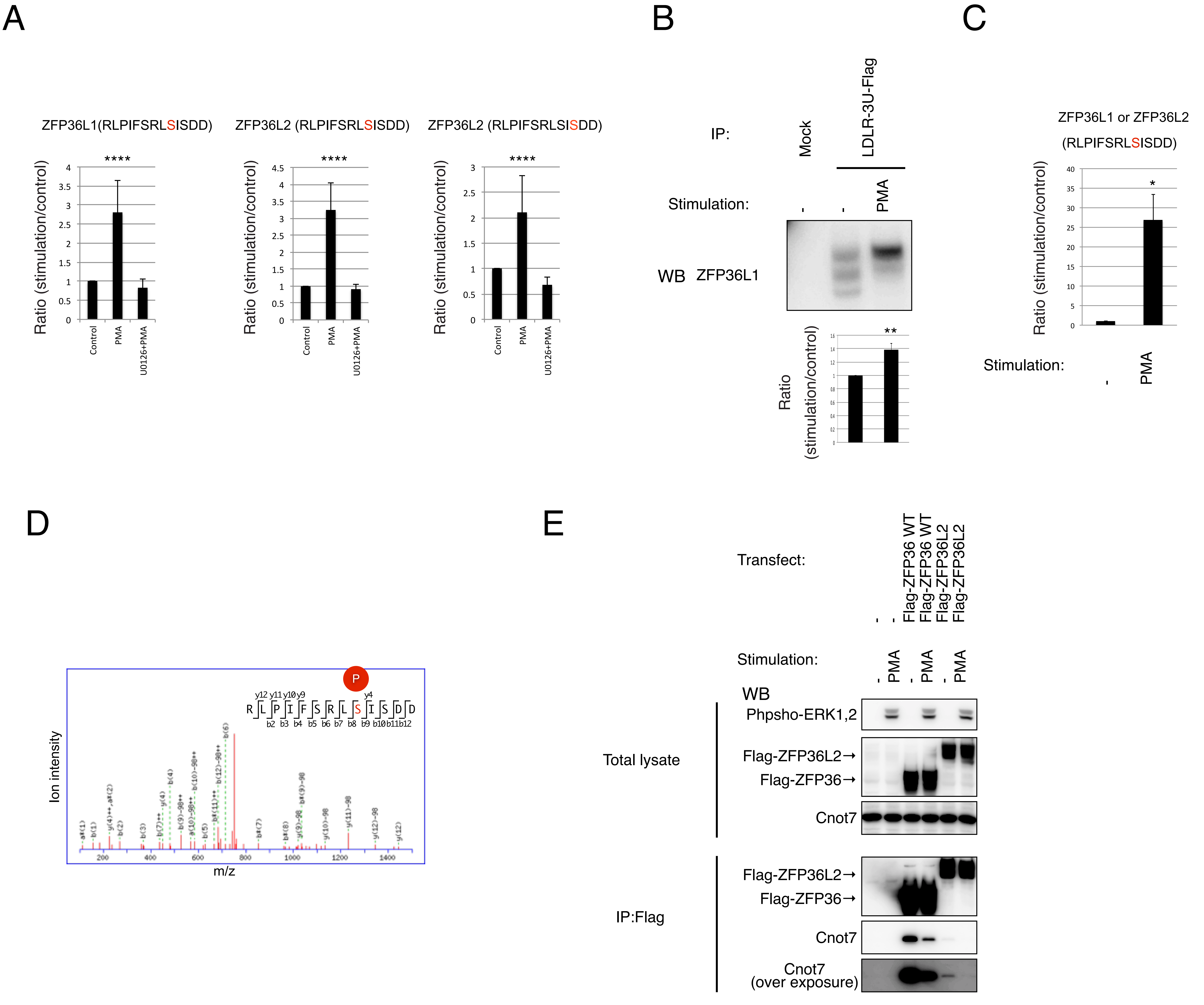

Adachi_et_al_FigS6

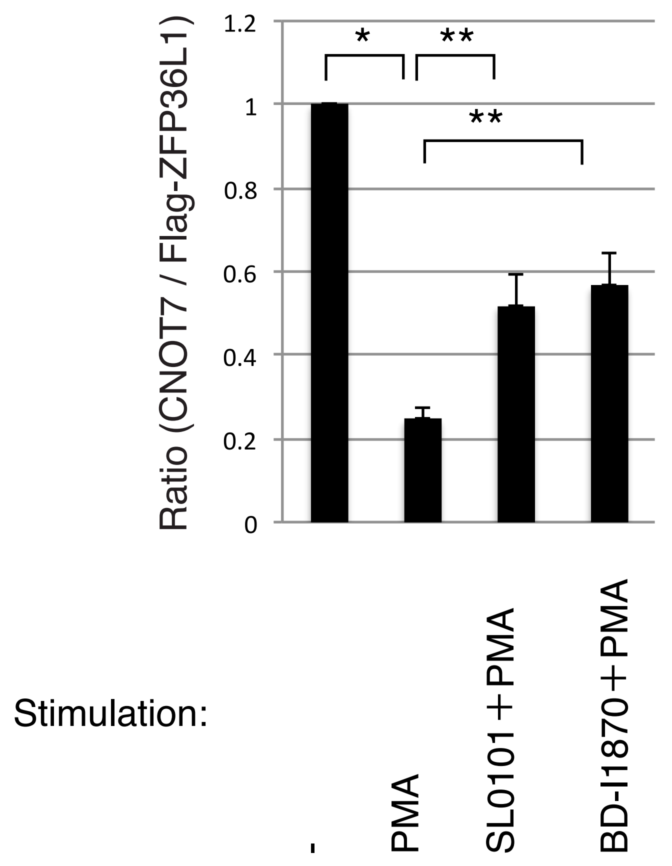

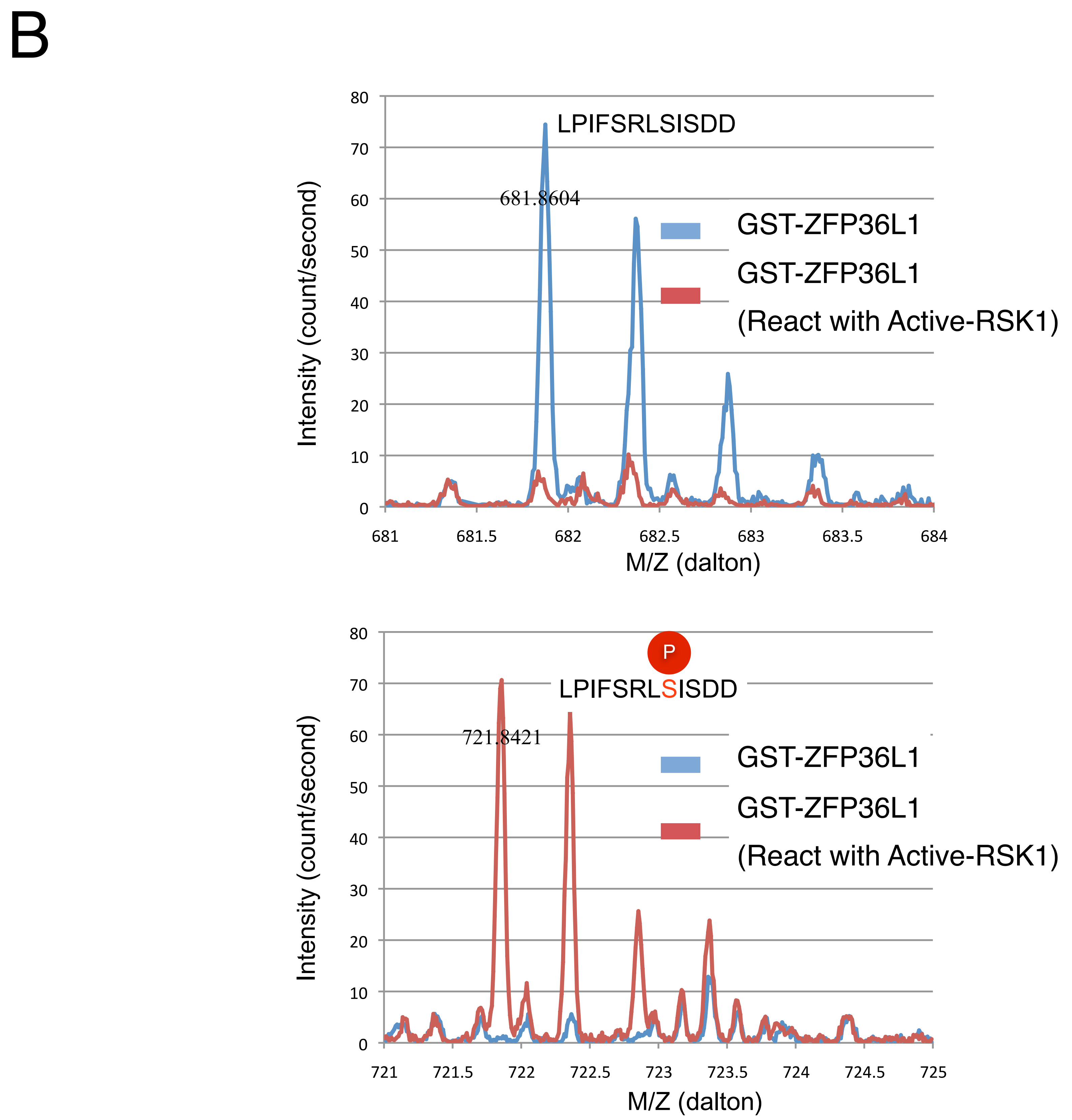

Adachi_et_al_FigS7

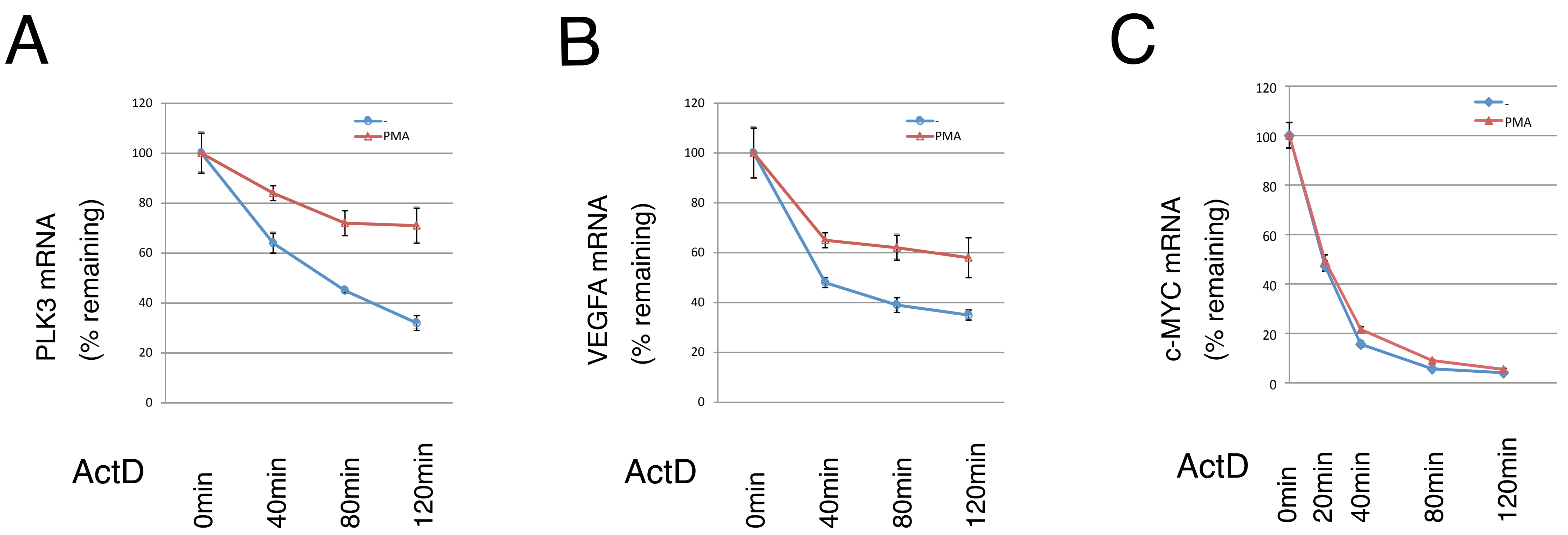

Supplement: SUPPLEMENTARY DATA [file supp_gku652_nar-02430-y-2013-File007.pdf]
